# Profiling of chromatin accessibility identifies transcription factor binding sites across the genome of *Aspergillus* species

**DOI:** 10.1186/s12915-021-01114-0

**Published:** 2021-09-06

**Authors:** Lianggang Huang, Xuejie Li, Liangbo Dong, Bin Wang, Li Pan

**Affiliations:** 1 School of Biology and Biological Engineering, South China University of Technology, Guangzhou Higher Education Mega Center, Guangzhou, 510006 China; 2 Guangdong Provincial Key Laboratory of Fermentation and Enzyme Engineering, South China University of Technology, Guangzhou Higher Education Mega Center, Guangzhou, 510006 China

**Keywords:** Filamentous fungi ATAC-seq, Chromatin accessibility, Footprints, Transcription factor binding sites, *Aspergillus* species

## Abstract

**Background:**

The identification of open chromatin regions and transcription factor binding sites (TFBs) is an important step in understanding the regulation of gene expression in diverse species. ATAC-seq is a technique used for such purpose by providing high-resolution measurements of chromatin accessibility revealed through integration of Tn5 transposase. However, the existence of cell walls in filamentous fungi and associated difficulty in purifying nuclei have precluded the routine application of this technique, leading to a lack of experimentally determined and computationally inferred data on the identity of genome-wide *cis*-regulatory elements (CREs) and TFBs. In this study, we constructed an ATAC-seq platform suitable for filamentous fungi and generated ATAC-seq libraries of *Aspergillus niger* and *Aspergillus oryzae* grown under a variety of conditions.

**Results:**

We applied the ATAC-seq assay for filamentous fungi to delineate the syntenic orthologue and differentially changed chromatin accessibility regions among different *Aspergillus* species, during different culture conditions, and among specific TF-deleted strains. The syntenic orthologues of accessible regions were responsible for the conservative functions across *Aspergillus* species, while regions differentially changed between culture conditions and TFs mutants drove differential gene expression programs. Importantly, we suggest criteria to determine TFBs through the analysis of unbalanced cleavage of distinct TF-bound DNA strands by Tn5 transposase. Based on this criterion, we constructed data libraries of the in vivo genomic footprint of *A. niger* under distinct conditions, and generated a database of novel transcription factor binding motifs through comparison of footprints in TF-deleted strains. Furthermore, we validated the novel TFBs in vivo through an artificial synthetic minimal promoter system.

**Conclusions:**

We characterized the chromatin accessibility regions of filamentous fungi species, and identified a complete TFBs map by ATAC-seq, which provides valuable data for future analyses of transcriptional regulation in filamentous fungi.

**Supplementary Information:**

The online version contains supplementary material available at 10.1186/s12915-021-01114-0.

## Background

There have been 339 species identified in the *Aspergilli* genus [1], and they are of broad interest because of their industrial applications, importance as pathogens for animals and crops, and relevance for basic research. *Aspergillus* section Nigri is the fungal genus with the most sequenced genomes, as genomes from 27 species are already publicly available [2], and genomic analyses have led to a better understanding of fungal biology and improvements in the industrial use of these organisms [1, 3, 4]. Gene regulation is of major way by which fungi physiologically act in environmental circumstances and respond to changing conditions [5, 6]. However, in comparison with *Saccharomyces cerevisiae*, the knowledge of genome-wide regulatory mechanisms in *Aspergilli* is lagging. Transcription factors (TFs) are key regulators of biological processes that function by binding to transcriptional regulatory regions to control the expression of target genes. The *cis*-regulatory elements (CREs) related to TF binding play pivotal roles in the regulation of gene expression, and each TF recognizes a collection of similar DNA sequences, known as binding site motifs [7, 8]. Therefore, the ability to identify these CREs and TF motifs throughout *Aspergillus* genomes is an important step in understanding the regulatory functions of TF binding and gene expression. Analysis of genome sequences has revealed an average of 600 putative transcription factors in each *Aspergillus* fungus genome [3, 9]. Nevertheless, approximately 5% of the TFs in the *Aspergillus* genus have only been identified and not been studied further [3, 9]. Most genome-wide TFBs in the *Aspergillus* genome have not been experimentally determined or computationally inferred, and the sites remain unknown.

The gold-standard method for identifying in vivo CREs for TFs of interest is chromatin immunoprecipitation DNA sequencing (ChIP-seq) [10]. However, the scarcity of sequence-specific antibodies for most *Aspergillus* TFs or tagged target proteins for the more than 600 TFs in *Aspergillus* has prevented the widespread application of this method in *Aspergillus* [11]. So far, only a few ChIP-seq experiments have used epitope-tagged or customized TF antibodies to study *Aspergillus* TFs, including CrzA [12], SrbA [13], MetR [14], and AtrR [15] of *Aspergillus fumigatus*; WetA of *Aspergillus nidulans* [16]; CRE-1 of *Neurospora crassa* [17]; Tri6 of *Fusarium graminearum* [18]; *MoCRZ1* of *Magnaporthe oryzae* [19] and some chromatin modification such as H3K4 trimethylation in *A. nidulans* [20, 21]. These findings reveal that the available filamentous fungi ChIP-seq data are not abundant and performing ChIP-seq experiments in filamentous fungi is full of challenges. Additional high-throughput sequencing methods, i.e., DNase-seq and formaldehyde-assisted isolation of regulatory elements with sequencing (FAIRE-seq) combine enzymatic digestion of isolated chromatin with sequencing, were developed to identify CREs on a genome-wide scale [22, 23]. The shortcomings of these methods are that they require millions of cells as starting material, complex and time-consuming sample preparations, and many potentially loss-prone steps, such as adaptor ligation, gel purification, and cross-link reversal [24]. For these reasons, the DNase-seq analysis that we previously carried out in *Aspergillus oryzae* could not be effectively replicated [23]. Therefore, the development of feasible and scalable methods is required to facilitate the identification of regulatory elements.

Assay for transposase accessible chromatin sequencing (ATAC-seq) is a recently developed technique used to identify accessible regions and DNA footprints [25, 26]. Tn5 transposase integrates sequencing adapters directly into DNA, eliminating the need for multiple reactions and purification steps typically required for the construction of a sequencing library. As a result, significantly lower amounts of starting nuclei are required for the investigation of CREs. ATAC-seq is now routinely being applied to systematically identify *cis-*regulatory regions and DNA footprints in humans [27], mice [28], zebrafish [29], *Drosophila* [30], *Caenorhabditis elegans* [31], *Arabidopsis thaliana* [32], *S. cerevisiae* [33], and so on. However, in *Aspergillus* species, the existence of cell walls and the purification of nuclei preparation prevented the routine application of this technique. To overcome this obstacle, we performed an ATAC-seq assay for *Aspergilli* by protoplast preparation under cultivation conditions. We combined ATAC-seq and RNA-seq of different *Aspergillus* species and TF mutants to profile chromatin accessibility and identify gene regulatory elements in vivo on a genome-wide scale. Furthermore, we characterized the identified CREs in vivo by the synthetic minimal promoter driving expression of the reporter gene. Collectively, this study is an important step towards the meticulous analysis of industrial microbial transcriptional regulatory networks and provides valuable resource for future research aimed at characterizing or using gene regulatory elements.

## Results

### The ATAC-seq assay in the filamentous fungus

**Fig. 1:**
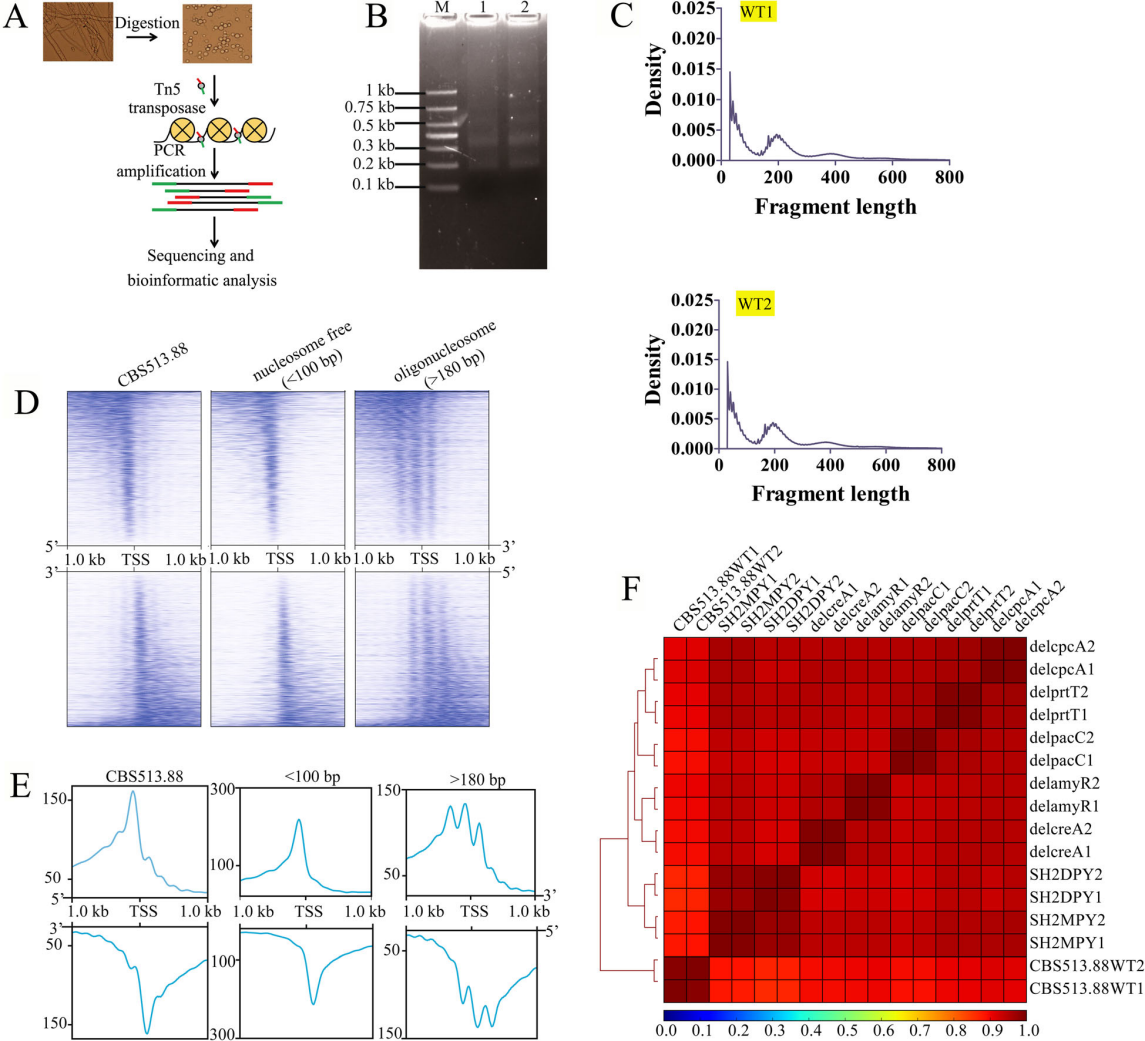
Schematic illustration of the Assay for Transposase-Accessible Chromatin with high-throughput sequencing (ATAC-seq) in *Aspergillus niger*. **a** Protoplasts used for nuclei isolation and schematic procedure of ATAC-seq technique. Protoplasts were prepared under the cultivation condition by adding lywallzyme. After filtration via miracloth membrane, the concentration of extracted protoplasts reached 10^5^/μL. **b** Fragment sizes for amplified ATAC-seq libraries by 15 cycles of PCR reaction. 5 × 10^4^ protoplasts of *A. niger* CBS513.88 were used for Tn5 transposase reaction, respectively. Lane 1: replicate 1, WT1; Lane 2: replicate 2, WT2. **c** Insert sizes determined by high-throughput sequencing. Peaks with different fragment length indicate that the fragments contain one or more nucleosomes. **d** Heatmaps showing the distribution of accessible regions around TSSs in *A. niger* CBS513.88: The meaning of “ < 100” is that the fragment sizes of pair-end reads were less than 100 bp of fragment size. And the meaning of “ > 180” is that the fragment sizes of pair-end reads were more than 180 bp. Regions of < 100 bp were assigned as nucleosome free and those of > 180 bp were assigned as oligonucleosomal [27]. The signals are derived from two technical replicates. **e** Read coverage of the 1-kb region flanking TSSs in *A. niger* CBS513.88. **f** Heatmap clustering of Spearman correlation coefficients for 16 *A. niger* ATAC-seq datasets

To identify chromatin accessibility and regulatory elements in filamentous fungi, we developed an ATAC-seq protocol for *Aspergillus* species by adding a step that released native nuclei by detergent lysis of protoplasts prior to the transposition step (Fig. 1a). A total of 5 × 10^4^ protoplasts were subjected to the extraction of native nuclei in lysis buffer containing 0.05% IGEPAL CA-630 (Sigma, I8896). After Tn5 transposition, PCR and electrophoresis were performed to detect the discrete nucleic acid bands (Fig. 1b). The insert size distribution of sequenced fragments from *A. niger* chromatin had a clear periodicity of approximately 200 bp (Fig. 1c, Additional file 1: Figure S1), suggesting many fragments are protected by integer multiples of nucleosomes.

We analyzed the pattern of mapped ATAC-seq reads around the transcription start site (TSS) in *A. niger* CBS513.88. Heatmaps showed the intensity of mapped ATAC-seq reads flanking 1-kb upstream and downstream of annotated TSSs in the AspGD database (Fig. 1d). Furthermore, separate heatmaps showed that fragments less than 100 bp clustered immediately upstream of TSSs throughout the *A. niger* genome. Fragments between 180 and 247 bp, corresponding to nucleosome footprints, were depleted from TSSs throughout the genome. Overall, the identified accessible regions were mostly enriched around the TSSs (Fig. 1d), which is consistent with the fact that these regions usually contain active regulatory *cis-*elements. The signal intensity of accessible regions showed a clear depletion and periodic peaks as the distance from the TSS (Fig. 1e), which is consistent with the presence of a positioned nucleosome + 1 that serves as barrier, dictating the positioning of neighboring nucleosomes (especially nucleosome − 1). This phenomenon attenuates at further distances from the barrier [34]. Furthermore, using ATAC-seq for *A. niger*, we generated paired-end ATAC-seq libraries for 16 samples, and the libraries ranged from 35.9 to 104.9 million reads (Additional file 2: Table S1). The percentages of reads in two replicate samples of CBS513.88_WT1 and CBS513.88_WT2 that aligned to the *A. niger* CBS513.88 genome was 84.23% and 86.31%, respectively (Additional file 2: Table S1). The insert size distributions of sequenced fragments from 16 ATAC-seq libraries of *A. niger* had a similar periodicity (Additional file 1: Figure S1). Spearman correlation analysis was used to inspect the reproducibility of biological and technical replicates. Heatmap clustering of Spearman correlation coefficients for 16 *A. niger* ATAC-seq datasets revealed a strong correlation between replicates of the same strain or the mutant and a lower correlation between different strains (Fig. 1f). Taken together, these data suggested that the ATAC-seq protocol of filamentous fungus *A. niger* could provide data on accessible regions of chromatin in a genome-wide search for regulatory elements.

### Identification of genome-wide chromatin accessibility and comparison of open chromatin profiles among *Aspergillus* species and strains

**Fig. 2:**
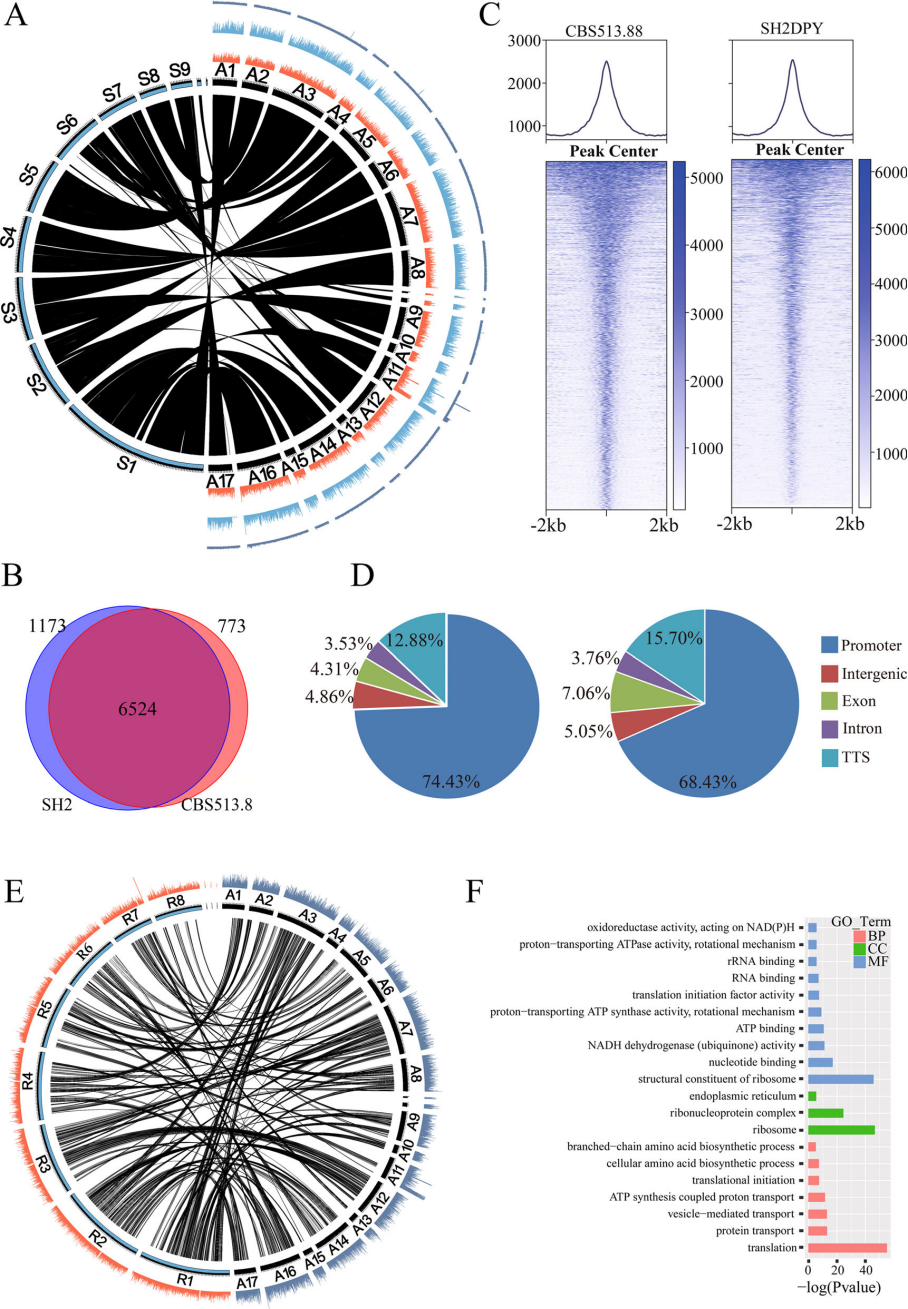
Whole-genome landscape of chromatin accessibility in *A. niger*. **a** Syntenic analysis between *A. niger* CBS513.88 (contig A1-A17 corresponding to An01-An19) and SH2 (contig S1-S11), and visualization of ATAC-seq peak signals in *A. niger* CBS513.88 (orange) and SH2 (blue). The outer signal represents the *A. niger* CBS513.88 naked genomic DNA which was used as the integration control of Tn5 transposase. **b** A Venn diagram showing the overlap of accessible regions between *A. niger* CBS513.88 and SH2. **c** Density plots of mapped reads (upper) and heatmap of peak length (bottom) for the 2-kb region flanking the peak center. **d** Distribution of accessible regions in the promoter, intergenic region, exon, intron, and TTS in *A. niger* CBS513.88 and SH2. **e** Syntenic analysis of ATAC-seq peaks between *A. niger* CBS513.88 (contig A1-A17) and *A. oryzae* niaD30 (contig R1-R8). **f** Functional clustering of overlapping ATAC-seq peaks of *A. niger* CBS513.88 and *A. oryzae* niaD300

Visualization of the *A. niger* CBS513.88 and SH2 ATAC-seq data sets on a genome scale revealed that they were similar to each other, and the Tn5 integration regions of naked genomic DNA were uniformly distributed along the entire chromosome (Fig. 2a). The syntenic analysis showed that most of the genomic sequences were homologous between *A. niger* strains CBS513.88 and SH2, except for a missing DNA fragment in *A. niger* CBS513.88 contig VIII_An18 and a fragment in *A. niger* SH2 contig S6 (Fig. 2a). A total of 7297 and 7697 accessible chromatin regions (peaks) were identified in *A. niger* CBS513.88 and SH2, respectively (Fig. 2b and Additional file 3: Table S2). Among these accessible regions, 6524 regions overlapped, indicating that these regions contained common *cis-*elements for both strains. These common regulatory elements might play a basal transcriptional regulatory role in *A. niger* species. Additionally, 773 and 1173 accessible regions are specific for *A. niger* CBS513.88 and SH2, respectively, and these accessible regions contained differential *cis-*elements for each strain. A heatmap of the peak signal for the 2-kb flanking region of the peak center (Fig. 2c) showed that most of the accessible regions formed sharp peaks in the center and ranged from narrow to wide, which provided valuable data depending on *cis*-element motifs in these accessible regions. On the genome scale, accessible regions are highly enriched in promoter regions (Fig. 2d), which was consistent with the distribution of accessible regions in Fig. 1d. In addition to promoter regions, the transcription terminator site (TTS) regions contain the maximal accessible regions (12.88% for CBS513.88 and 15.70% for SH2, Fig. 2d). These accessible regions in TTSs indicated that gene downstream regions also contain regulatory elements for gene transcription.

As the GRAS strains, *A. niger* and *A. oryzae* share the common characteristics for the production of heterologous protein, protease, and natural products. To understand the syntenic orthologues of accessible regions across the *Aspergillus* species, we compared the *A. niger* CBS513.88 and *A. oryzae* niaD300 ATAC-seq peaks on a genome-wide scale. Our results showed that these two *Aspergillus* species had strong peak synteny, and there were 1564 syntenic orthologues of ATAC-seq peaks identified between *A. niger* CBS513.88 and *A. oryzae* niaD300 (Fig. 2e, Additional file 4: Table S3). Then, we located the target genes of these syntenic peaks and analyzed their GO enrichment function (Fig. 2f). Ribosomes related to GO categories were significantly enriched in these target genes, especially for the following three categories: “structural constituent of ribosome (MF),” “ribosome (CC),” and “translation (BP).” These highly enriched target genes indicated that *Aspergillus* species strains possessed the highly homologous regulatory mechanisms in terms of protein synthesis. Therefore, ATAC-seq data can be used for comparative genome analysis to explain the conservation of the evolutionary process of organisms at the genome level.

### Identification of significantly changed chromatin accessibility driven by transcription factors and chromosome regulatory factor in *Aspergillus* species

**Fig. 3:**
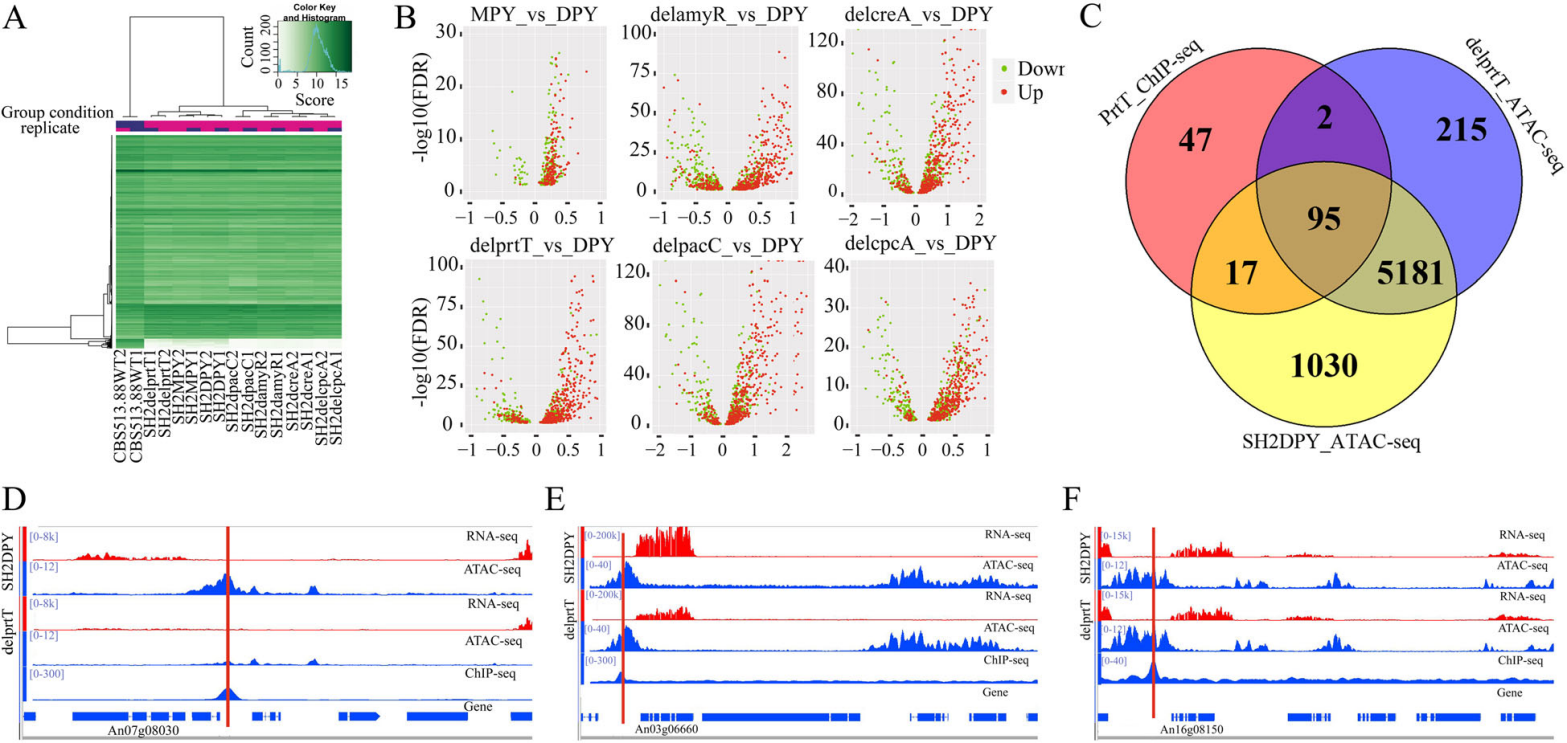
Differential open chromatin accessibility analysis of *A. niger*. **a** Clustering analysis of ATAC-seq peaks for 16 *A. niger* samples. **b** Volcano plots of chromatin accessibility change combined with its annotated genes. ATAC_Fold means an increase (> 0) or decrease (< 0) in chromatin accessibility (*X*-axis). *Y*-axis represents the confidence coefficient of ATAC_fold. The red and green color points represent up- and downregulated genes with significance (|log2(Fold_Change)| ≥ 1, *P* < 0.05), respectively. **c** The overlap of peaks between PrtT ChIP-seq and ATAC-seq. **d–f** Visually display of the coupling analysis of ATAC-seq, ChIP-seq, and RNA-seq using IVG browser. The red vertical line indicates the PrtT binding site. The An number of target genes were labelled in the corresponding position

A clustering map of ATAC-seq peaks for 16 *A. niger* samples indicates that there were common ATAC-seq patterns among all *A. niger* strains and specifically showed that SH2 and its derivatives did not have ATAC-seq signals corresponding to the 200-kb missing genomic DNA fragments (Fig. 3a) [4]. Furthermore, we identified the ATAC-seq peaks that significantly changed accessibility (FDR < 0.05) among *A. niger* SH2 cultured under different conditions and its TF-deleted strains (Fig. 3b and Additional file 5: Table S4). A total of 4009 of the differential ATAC-seq peaks (DAPs) in the promoter regions were identified when comparing with *A. niger* SH2 cultured in DPY and MPY media (Additional file 5: Table S4). We found that changing the culture conditions could slightly alter the chromatin accessibility, in which 3853 of the DAPs were specific to *A. niger* SH2 cultured under MPY medium (Additional file 5: Table S4). Almost all upregulated genes were driven by the MPY-specific ATAC-seq peaks (Fig. 3b: MPY_vs_DPY). Besides, the induced conditions such as alternative carbon sources or differential pH could also slightly alter the chromatin accessibility (Data submitted to NCBI, PRJNA692847), and low-level CreA can strongly inhibit the expression of *xlnD* and *abfA* (Additional file 1: Figure S2). Furthermore, we identified TF-specific chromatin accessibility using the peak signal intensity of *A. niger* SH2 compared with that observed in TF-deleted strains (FDR < 0.05). In total, 1641 of the amyR-specific peaks, 1156 of the prtT-specific peaks, 773 of the cpcA-specific peaks, 1301 of the pacC-specific peaks, and 1305 of the creA-specific peaks in the promoter regions exhibited significantly higher accessibility (FDR < 0.05) than those in the corresponding TF-deleted strains (Additional file 5: Table S4). The distributions of upregulated genes were unbalanced and trended towards TF-specific chromatin accessibility (Fig. 3b).

To confirm the ATAC-seq data, we performed ChIP-seq of the protease regulator PrtT (NCBI accession NO.: PRJNA692789). A total of 161 specific peaks were enriched, of which 97 (60.24%) peaks could be mapped with delprtT and 112 (69.57%) peaks with WT (Fig. 3c). The conserved motif CCGHCGG [35] could also been enriched (Additional file 1: Figure S3). Furthermore, the coupling analysis of ChIP-seq, RNA-seq, and ATAC-seq data of PrtT (Fig. 3d–f and Additional file 1: Figure S4) showed three major regulation categories of TF: (i) the deletion of PrtT leads to decreased chromatin accessibility, thereby downregulating target genes (Fig. 3d and Additional file 1: Figure S4a); (ii) the deletion of PrtT does not affect chromatin accessibility, but downregulates target genes (Fig. 3e and Additional file 1: Figure S4b); (iii) the deletion of PrtT neither affects chromatin accessibility, nor affects gene expression (Fig. 3f and Additional file 1: Figure S4c).

**Fig. 4:**
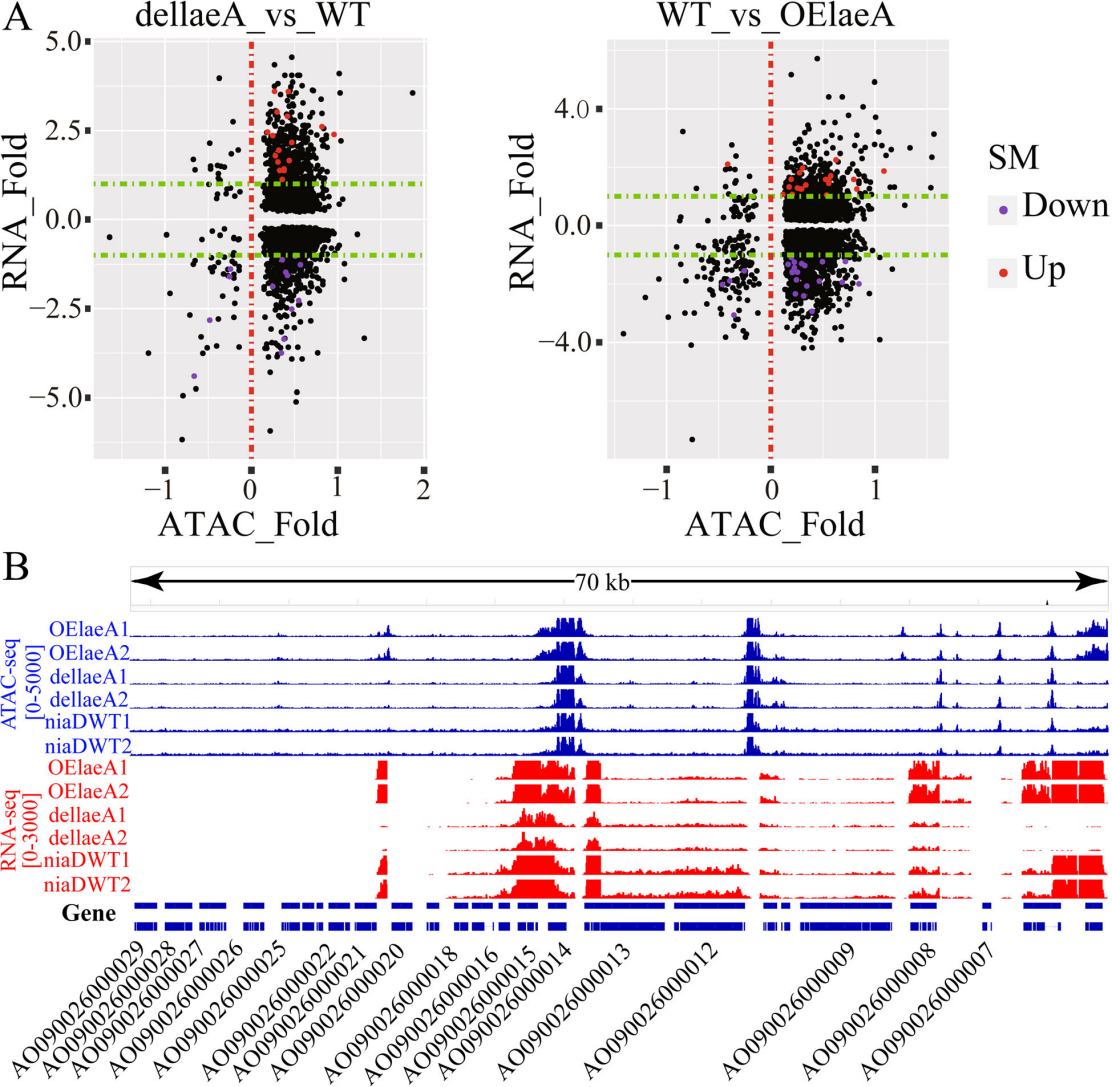
Effect of LaeA on the activity of secondary metabolism gene cluster in *A. oryzae*. **a** Scatter plot of differentially expressed genes (*P* < 0.05) and chromatin accessibility between *A. oryzae* WT strain and *laeA* mutants. Significantly differentially expressed SM genes (|log2(Fold_Change)| ≥ 1, *P* < 0.05) are indicated by red and purple dots. The green dotted line indicates |log2(Fold_Change)| = 1. All data are derived from two technical replicates. **b** A representative aflatoxin biosynthetic pathway in *A. oryzae* showing a comparison between ATAC-seq and RNA-seq for samples of *A. oryzae* WT strain and *laeA* mutants. OEleaA1, OElaeA2, dellaeA1, and dellaeA2 were biological replicates in RNA-seq and ATAC-seq analysis for the *A. oryzae laeA* mutants (OElaeA and dellaeA)

The biosynthetic gene clusters of secondary metabolites (SMs) in *Aspergillus* species are frequently silent or inactive under normal laboratory conditions due to a tendency to be located in subtelomeric regions with a high degree of heterochromatin condition; however, these genes can be activated by changing the chromosomal state of the SM gene clusters from heterochromatin to euchromatin [36]. LaeA has been acknowledged as a global regulator of secondary metabolism in *Aspergilli*. Deletion of LaeA in filamentous fungi decreased the virulence of the strains and the production of secondary metabolites. And it has been reported that deletion or overexpression of LaeA could be used to explore numerous secondary metabolite clusters [37–39], although the physiological function of LaeA in modulating gene expression remains little known [40]. To investigate whether LaeA could regulate the chromatin accessibility in *Aspergilli*, we constructed ATAC-seq libraries of dellaeA and OElaeA and identified the ATAC-seq peaks with significantly changed accessibility (FDR < 0.05) in dellaeA-vs-WT (4725 DAPs) and OElaeA-vs-WT (5572 DAPs). (Figure 4a, Additional file 1: Figure S5; Additional file 2: Table S1, and Additional file 6: Table S5). SM biosynthetic gene clusters in the *A. oryzae* RIB40 genome contain 621 biosynthetic SM genes predicted by Secondary Metabolite Unique Regions Finder (SMURF) [41]. The chromatin regions in front of 138 and 164 of the SM biosynthetic genes in *A. oryzae* OElaeA and dellaeA mutants significantly changed accessibility (FDR < 0.05) to induce significant regulation of 42 and 32 SM biosynthetic genes, respectively (Fig. 4a and Additional file 6: Table S5). The 54 backbone genes of SM biosynthesis include polyketide synthases (PKSs) and non-ribosomal peptide synthetases (NRPSs) from the *A. oryzae* RIB40 genome; only 12 backbone genes of SM biosynthesis significantly changed accessibility (FDR < 0.05) to induce significant regulation of the corresponding SM backbone genes in OElaeA and dellaeA mutants, which contained 7 NRPSs, 4 PKSs, and 1 DMAT (Additional file 6: Table S5). We found that the knockout and overexpression of the *laeA* gene only up- and downregulated the SM biosynthetic genes that were expressed in the wild-type strain, while there was never de novo activation of those SM biosynthetic genes that were not expressed in the wild-type strain (Additional file 1: Figure S6). Furthermore, we investigated chromatin accessibility and transcriptional activity in the famous aflatoxin (AF) biosynthesis pathway of *A. oryzae* (Fig. 4b). The sequences of the aflatoxin (AF) biosynthesis gene clusters were divided into two parts: active and inactive chromatin regions. The sequences from AO090026000004 to AO090026000020 were the active chromatin regions, which were characterized by highly expressed biosynthesis genes. The overexpression and knockout of the *laeA* gene were able to change the chromatin accessibility in the active regions. The key transcription factor *aflR* (AO090026000014) was in the region of high chromatin accessibility, while the coactivator *aflJ* (AO090026000015) became highly expressed following the overexpression of *laeA*. The other sequences from AO090026000020 to AO090026000031 were in inactive chromatin regions in which no ATAC-seq peaks were identified, and biosynthesis genes had no expression or the lowest level of expression. The AF biosynthesis pathway components were located in a laeA-dependent SM biosynthetic gene cluster. Taken together, the reason for the lack of AF biosynthesis in *A. oryzae* might be inactive chromatin regions around the AF biosynthesis pathway.

### Identification of footprints in filamentous fungus *Aspergillus niger*

**Fig. 5:**
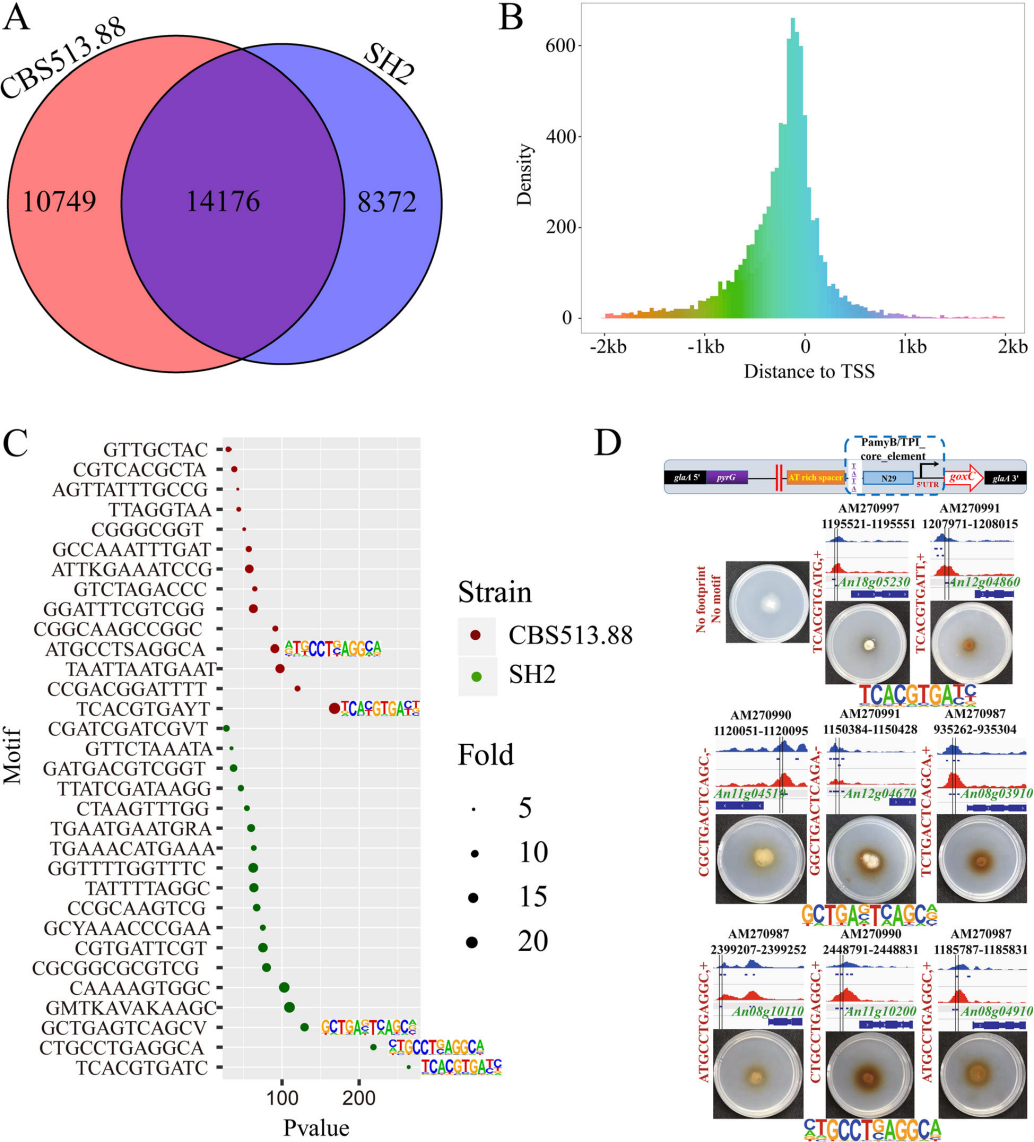
Identification of de novo predicted footprints in *A. niger* CBS513.88 and SH2, and functional verification in regulating gene expression. **a** Venn diagram analysis of identified footprints in *A. niger* CBS513.88 and SH2. **b** Statistical analysis of the distances between the identified DNA footprints and the TSSs. **c** De novo prediction of transcription factors in *A. niger* CBS513.88 (red) and SH2 (green)*.* The circle size represents the enrichment fold. The top three transcription factor motifs in *A. niger* SH2 are labeled with the corresponding binding motifs. The *x*-axis represents *P* value. **d** In vivo functional verification of putative de novo transcription factor targeting sites were carried out by artificially synthesized minimal promoters. The red dividing line represents the location of the footprints to be verified. The red text on the side is the TF binding motif contained in the verified DNA instances. From top to bottom, there were 2 E-box of bHLH factors (motif TCACGTGATC), 3 core elements of bZIP factors and 3 SltA homologs binding motif (motif CTGCCTGAGGCA) target sites validated by phenotype plates in vivo

DNA regions of footprints bounded by DNA-binding proteins are protected from Tn5 integration, at which the read coverage suddenly drops [26, 42]. A high-depth ATAC-seq can be used to identify depleted narrow regions in open chromatin regions of a genome corresponding to a single TF footprint [26, 42, 43]. We used the “*wellington_footprints.py*” function of the pyDNase package [44] to systematically identify Tn5 integration-insensitive sites from CBS513.88 and SH2 and footprints from ATAC-seq peaks (Fig. 5a). In total, there were 24,925 and 22,548 DNA footprints detected in vivo across the *A. niger* genome from 7297 and 7697 ATAC-seq peaks of CBS513.88 and SH2, respectively (footprint data from all wild-type and TF-deleted strains can be found in Additional file 7: Table S6). A total of 14,176 DNA footprints overlapped between CBS513.88 and SH2 ATAC-seq data. The DNA footprints identified by the ATAC-seq data were enriched 1-kb upstream and 0.5-kb downstream of the TSSs (Fig. 5b). To further characterize genomic footprints, we performed de novo motif enrichment analysis on the footprints from CBS513.88 and SH2 using the “*findMotifsGenome.pl*” function of the HOMER package with the default settings [45]. A total of 15 and 17 de novo TFBs with a *P* value threshold of 1.0E−12 were enriched more than 5-fold from the two sets of footprint data from CBS513.88 and SH2, respectively (Fig. 5c and Additional file 8: Table S7). De novo TFBs of other SH2 TF-deleted strains with a *P* value threshold of 1E−50 were also identified using the HOMER method (Additional file 1: Figure S7 and Additional file 8: Table S7). The E-box of bHLH factors (motif TCACGTGATC), the SltA homolog binding motif (motif CTGCCTGAGGCA), and the core element of bZIP factors (motif GCTGAGTCAGCV) were among the top 5 most enriched motifs according to *P* value in all de novo TF binding motifs (Fig. 5c). Instances of each TF binding motif were identified using the “*annotatePeaks.pl* -m” function of the HOMER package to search for DNA sequences in the 24,925 DNA footprints from *A. niger* CBS513.88 and the 22,548 DNA footprints from *A. niger* SH2 (Additional file 9: Table S8). Instances of genes controlled by de novo TF binding at motifs were identified in footprints in the promoter regions, and they were analyzed for the functional enrichment of corresponding TF binding motifs (Additional file 1: Figure S8). In the *A. niger* SH2, 269 genes under the control of the de novo motif TCACGTGATC (E-box) were enriched in GO categories “structural constituent of the ribosome” and “translation” (Additional file 1: Figure S8a). In addition, 154 genes under the control of the de novo motif CTGCCTGAGGCA (SltA homolog) were enriched in the molecular function GO categories “ATP binding” and “structural constituent of the ribosome;” in the cellular components of “ribosome,” “mitochondrial inner membrane,” and “vacuole;”; and for the biological processes of “translation” and “proteolysis” (Additional file 1: Figure S8b). Then, 54 genes under the control of the de novo motif GCTGAGTCAGCV (bZIP factors) were also enriched in the molecular function GO categories “zinc ion binding,” “DNA binding,” and “transcription factor activity” in the nucleus for the process of regulation of transcription (Additional file 1: Figure S8c).

Furthermore, to detect whether the ATAC-seq footprints containing the TF binding motifs drove the expression of functional target genes, we constructed a minimal synthetic promoter consisting of a core element, an ATAT-binding box, an AT-rich spacer, and a footprint library-driven UAS element with a TF binding site motif (Additional file 1: Figure S9) [46]. A 29-bp sequence from the TSS in the *amyB* (An05g02100) promoter of *A. niger* was chosen as the core element due to the high-level expression of the *amyB* gene. The glucose oxidase *goxC* (An01g14740) was used as a reporter, the expression of which would be revealed by a colored colony. The repression under ER stress (RESS) element identified in our prior study [46] was used as a positive control, and it also produces colored colonies (Additional file 1: Figure S9). As a negative control, a sequence containing only the *amyB* core element, the ATAT sequence, and the AT-rich spacer was constructed (no colored colonies but basal transcription can occur (Additional file 1: Figure S9)). We used this minimal promoter system to determine whether the ATAC-seq footprints containing de novo motifs, such as motif TCACGTGATC (E-box), motif CTGCCTGAGGCA (SltA homolog), and motif GCTGAGTCAGCV (bZIP factors), could drive the expression of target genes (Fig. 5d). Verifying the translation and ribosome functions controlled by the motif TCACGTGATC (E-box), the footprint of An12g04860, which encodes a ribosomal protein of the large subunit L30, was found to drive the expression of the reporter gene in vivo, resulting in colored colonies (Fig. 5d). The footprints containing motif CTGCCTGAGGCA located in the promoter regions of An08g04910 and An11g10200, which encode the NADH-ubiquinone oxidoreductase and cytochrome C oxidase subunit in the electron respiratory chain, respectively, also drove the expression of the reporter gene in vivo. The footprints containing the motif GCTGAGTCAGCV, which is located in the promoter regions of An11g04510 and An12g04670 (related to translation release factor activity and translation initiation factor, respectively), were confirmed to drive the expression of the reporter gene in vivo. Taken together, genome-wide DNA footprints were systematically identified from accessible chromatin regions based on our ATAC-seq data. In addition, de novo motifs enriched from ATAC-seq footprints were able to drive the expression of their target genes in vivo*.*

### Known TF motifs of *Aspergillus niger* enrichment in ATAC-seq footprints

**Fig. 6:**
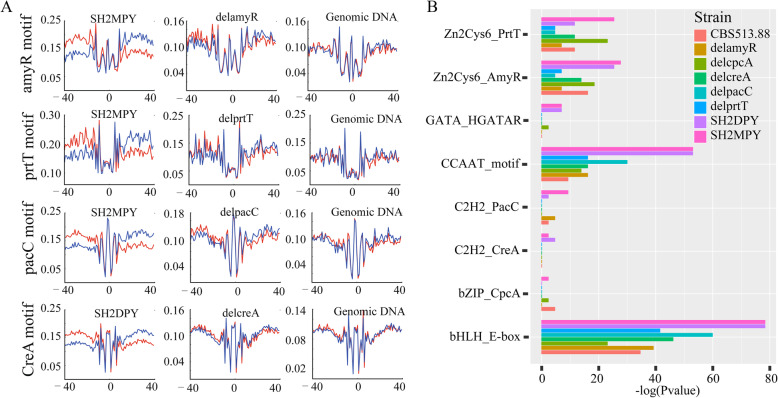
Distribution of Tn5 integration around known footprints and enrichment analysis of known TF motifs. **a** Distribution of Tn5 integration sites in the ATAC-seq data of SH2MPY (left), TF deletion strains (middle), and naked genomic DNA (right) around known motifs including *amyR*, *prtT*, *pacC*, and *creA*. Tn5 integration sites showed similar patterns around known TF motifs between TF deletion strains and naked genomic DNA whereas the patterns are different for SH2MPY. The red line indicates the integration sites on the positive-sense strand whereas the blue line indicates those on the anti-sense strand. *Y*-axis represents average Tn5 integrations. The frequency analysis was carried out by combining two technical replicates. **b** Enrichment analysis of known TF motifs in *A. niger* CBS513.88, SH2, and TFs deletion mutants; − log(*P* value) was used as a significant criterion

We determined the transposase Tn5 integration patterns around the binding sites of AmyR, PrtT, PacC, and CreA in the *A. niger* SH2 cultured under MPY medium (Fig. 6a), while these patterns for known TFs were also evident in naked genomic DNA (Fig. 6a). We further found that distributions of Tn5 integration sites in the flanking regions of binding site motif sequences were asymmetrical when comparing the anti-sense and sense strand and the patterns which were completely different from the symmetrical patterns observed in the naked genomic DNA. Furthermore, to elucidate whether strand-specific Tn5 integration patterns around TF binding sites are caused by protein/DNA interactions, we calculated the Tn5 integration frequency in TF-deleted strains (Fig. 6a). In this experiment, the conserved DNA motif should not be enriched, for there was no corresponding known TF to be expressed in vivo. Tn5 integration sites around the known TF binding sites in the absence of the corresponding TF condition showed a similar pattern to that of genomic DNA, which was a symmetrical integration pattern around TF binding sites on both DNA strands. The asymmetrical pattern of Tn5 integration around TF binding sites in our ATAC-seq data was distinctly different from the no TF binding pattern on both TF-deleted strains in vivo and naked genomic DNA in vitro (Fig. 6a). We demonstrated here that strand-specific Tn5 integration patterns around TF binding sites are caused by protein/DNA interactions, which could be used as a signal indicating an active TFBs.

To determine the enrichment of known TF binding motifs from ATAC-seq footprints by comparing ATAC-seq data from *A. niger* WT strain and its TF-deleted strains, we collected the available binding motif sequences of 19 known *Aspergillus* TFs [23] and employed the “findMotifsGenome.pl” function of the HOMER package to identify overrepresented motifs of known *Aspergillus* TFs from ATAC-seq footprint data. We found that the binding motifs of bZIP CpcA, C2H2 CreA, and PacC were not overrepresented in the ATAC-seq footprints of the corresponding TF-deleted strains (*P* value > 1.0E−2) (Fig. 6b). Although the binding motifs of the Zn_2_Cys_6_ family AmyR and PrtT were also enriched in the footprints from the TF-deleted strains delamyR and delprtT, respectively, the enrichment *P* values of AmyR and PrtT motifs were less than 1.0E−2 (Fig. 6b). The binding motifs for bHLH family E-box, CCAAT binding complex (CBC), Zn_2_Cys_6_ family AmyR, and PrtT were significantly enriched in all *A. niger* strains, especially the overrepresentative enrichment of SH2 in MPY medium (Fig. 6b). Other TF family motifs, such as bZip CpcA, GATA AreA, C2H2 CreA, and PacC, were only enriched in the ATAC-seq footprints of the SH2 cultured in MPY and DPY medium (Fig. 6b)*.* Footprints that contained a significant motif match were considered to be instances of predicted binding motifs. We used the “annotatePeaks.pl -m” function of the HOMER package to detect the motif instances of known TFs from footprints by comparing *A. niger* WT strains and the corresponding TF-deleted strains (Additional file 10: Table S9). The motifs of AmyR, PrtT, PacC, and CreA were identified in 765, 158, 821, and 2865 motif instances in the SH2 cultured in MPY, respectively (Additional file 10: Table S9). However, we still detected 593 instances of AmyR binding, 103 instances of PrtT binding, 656 instances of PacC binding, and 2648 instances of CreA binding from footprints in the TF-deleted strains delamyR, delprtT, delpacC, and delcreA, respectively (Additional file 10: Table S9).

**Fig. 7:**
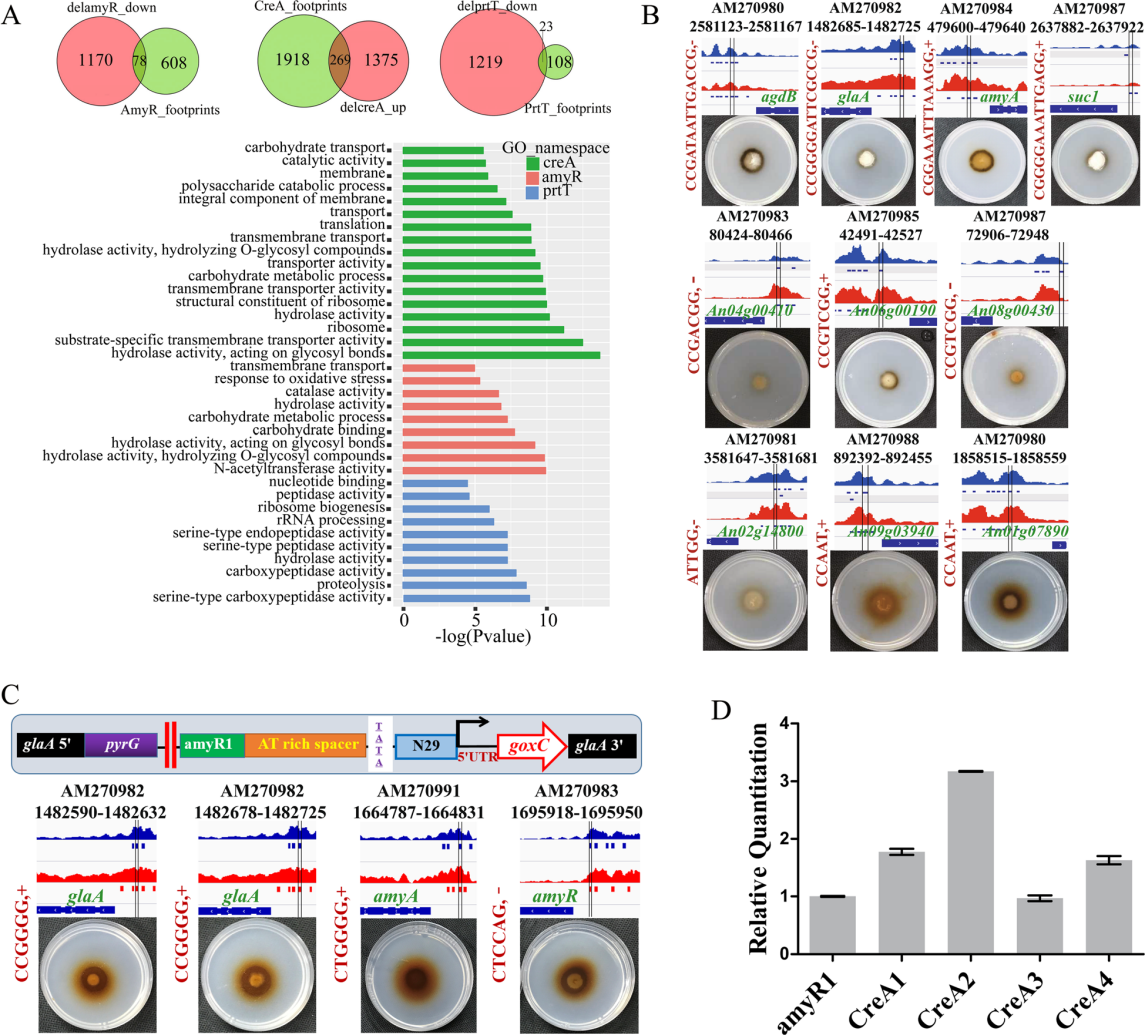
Functional analysis and verification of known TF motifs in ATAC-seq footprints. **a** Venn diagram analysis of TF binding motif instances of ATAC-seq footprints combined with RNA-seq data. Functional classification of the intersection genes of TF binding motif instances of ATAC-seq footprints combined with RNA-seq data through FungiFun. **b** In vivo functional verification of putative known transcription factor targeting sites was carried out by artificially synthesized minimal promoters. The red dividing line represents the location of the footprints to be verified. From top to bottom, there were 4 AmyR, 3 PrtT, and 3 CBC box (CCAAT) target sites validated by phenotype plates in vivo. **c** The repressors, CreA, were functionally verified by an artificial promoter containing AmyR instances. The active sites of 4 CreA were verified by phenotype plates, respectively. **d** qRT-PCR analysis of the *goxC* gene driven by CreA targeted instances. The experiment was performed in biological triplicate and *gpdA* served as the endogenous reference gene

Furthermore, to investigate whether instances of TF binding drove the expression of target genes, we used TF binding motif sites in ATAC-seq footprints combined with RNA-seq data for the changes in gene expression between the SH2 cultured in MPY and the corresponding TF-deleted strains (Fig. 7a). The expression of 1248 genes in the delamyR and 1242 genes in the delprtT was downregulated compared to what was seen in the control of the SH2 strain (Fig. 7a). Only 78 and 23 genes were downregulated in the delamyR and delprtT strains overlapped with motif target genes, respectively (Fig. 7a). As expected, 23 target genes under the control of PrtT were mainly involved in serine-type peptidase activity, and 78 target genes under the control of AmyR were functionally enriched in hydrolase activity, acting on glycosyl bonds (Fig. 7a). A total of 1644 genes were upregulated in the delcreA compared with the control of the SH2 strain, in which 269 genes overlapped with motif target genes from CreA (Fig. 7a). GO functional enrichment analysis revealed that 269 genes under the control of CreA were primarily involved in the molecular function of hydrolase activity and transporter activity. Besides, the enriched genes were clustered in the structural constituents of the ribosome, the cellular components of membranes and ribosomes, and their biological processes involved in carbohydrate catabolism, carbohydrate transport, and translation (Fig. 7a).

To experimentally assess whether footprints with these known TF binding motifs that were identified by ATAC-seq data were functional TF binding sites, we performed footprint-driven reporter assays in vivo (Fig. 7b). Three footprints containing the AmyR binding motif were located in promoters of glycosyl bond hydrolase genes: An01g10930 (*agdB*), An03g06550 (*glaA*), and An05g02100 (*amyA*). These AmyR binding motifs drove the expression of the reporter gene, resulting in colored colonies. Three footprints containing the PrtT binding motif in the promoter of peptidase genes, including An04g00410 (dipeptidyl peptidase III), An06g00190 (Sedolisin family secreted protease), and An08g00430 (serine-type carboxypeptidase), also drove the expression of the reporter gene. In addition, footprints containing CCAAT box or AreA binding motifs can also function as transcription activator (Fig. 7b and Additional file 1: Figure S10).

In addition to identifying the activation function of ATAC-seq footprints containing the known TF binding motifs, we further investigated whether ATAC-seq footprints containing repressor binding motifs could downregulate the expression of target genes (Fig. 7c). We constructed a minimal synthetic promoter with the footprint-driven UAS elements containing the AmyR binding motif instance (An01g10930, *agdB*) as a positive control (Fig. 7c). The footprint containing the repressor binding motif was assessed by assembling downstream of the AmyR footprint. *A. niger* CreA (An02g03830, contains two Cys_2_His_2_ zinc finger motifs) is the transcriptional regulator that mediates carbon catabolite repression [47]. However, our results showed that footprints containing CreA binding motif sites located in promoters of glycosyl bond hydrolases such as *glaA* (An03g06550), *amyA* (An12g06930), and *amyR* (An04g06910) were able to upregulate the expression of the reporter gene (Fig. 7c, d). Two footprints containing the CreA binding motif in the *glaA* promoter possessed the same ability to activate functional gene expression (Fig. 7c). Taken together, the transcriptional regulator CreA acts not only as repressor but also as activator.

## Discussion

In ATAC-seq, the Tn5 transposase integrates its adaptor payload into locations of open chromatin, which can be amplified by PCR and assessed with high-throughput sequencing to study protein/DNA interactions on a genome scale [27]. Here, we developed an ATAC-seq protocol for filamentous fungi to identify chromatin accessibility on a genome scale. Our initial attempts failed to generate ATAC-seq data from the chromatin following digestion of Tn5 transposase, harvesting the mycelium and reducing it to powder by grinding it in liquid nitrogen with a mortar and pestle. The gel image analysis shows no discrete nucleic acid bands, and this method resulted in a high noise-to-signal ratio after sequencing. In our modified *Aspergillus* ATAC-seq protocol, the lysis of the *Aspergilli* cell wall was carried out by adding hydrolase during cultivation, and protoplasts were obtained by filtration through a miracloth membrane. High-quality intact nuclei released from *A. niger* protoplasts, which are similar to mammalian cells, were used to improve the data collected from ATAC-seq. ATAC-seq requires few cells, so a sufficient number of protoplasts were easily obtained from *A. niger* cultured under various conditions. Yeast spheroplasts from *S. cerevisiae* and *Schizosaccharomyces pombe* have also been used in ATAC-seq assays [33]. Furthermore, protoplast preparation could occur with *A. niger* cells cultured under various conditions to decrease experimental variation. In our *A. niger* ATAC-seq library, we detected the same periodicity pattern in the insert size distribution of sequenced fragments from chromatin as what was observed in a human lymphoblastoid cell line [27] and in *Arabidopsis thaliana* [43]. The *A. niger* ATAC-seq library has a high signal-to-noise ratio, which was controlled by comparing the data to Tn5 integration into naked genomic DNA. The reproducibility of biological and technical replicates in our *A. niger* ATAC-seq protocol was relatively high. In ATAC-seq experiments using mammalian cells and plant cells, typically ~ 30–70% of the sequenced reads are aligned to the organellar genomes [27, 43]. In our *A. niger* CBS513.88 ATAC-seq results, a significantly higher fraction of reads were mapped to the nuclear genome. These data show that our *Aspergillus* ATAC-seq protocol is an effective method that can be applied to the analysis of other species of filamentous fungi.

The chromatin accessibility regions of *A. niger* CBS513.88 and SH2 were similarly distributed across the various elements of the genome and showed a high proportion at TSSs (~ 70%) and a low proportion at intergenic regions (~ 5%), which was contrary to humans [48], Arabidopsis [32], and tomato [8]. This difference may reflect the smaller size of the *A. niger* genome (35 Mb) [3] compared with the larger and less compact genomes of humans and plants. In addition, TTS also contained a small number of accessibility regions (~ 13%), which is similar to what was observed in the mouse [28]. Therefore, we reasoned that TTSs may also play an important role in the transcriptional regulation of genes and downstream translational control in *A. niger*.

Prior works found that the accessible chromatin regions were positively correlated with gene expression changes of the nearest target genes [28, 32, 49]. However, the regulatory regions could also be suppressed by some TFs or nucleosomes [49]. Our results further verified the coexistence of transcriptional activation and inhibition in the accessible region. Besides, the gene transcription activation can be accomplished by a single TF alone, or by a combination of multiple TFs (Fig. 3). Under the condition of single activation, TF-deleted directly affects chromatin accessibility (Fig. 3d and Additional file 1: Figure S5a). For multi-gene regulatory regions, there is a competitive regulation of TFs [50, 51]. When TF is missing, other proteins occupy the vacated sites, thereby maintaining chromatin accessibility. In this case, the transcription of downstream gene is jointly determined by the lack of TF and the newly occupied TFs.

Comparative genomics focuses on the comparison of DNA coding regions between different species, revealing the eukaryotic genomic evolution of different *Aspergillus* species [9]. In comparing open chromatin regions (noncoding sequences) between *A. niger* and *A. oryzae* species, we discovered that syntenic orthologues of 1564 accessible regions across *A. niger* and *A. oryzae* drive the expression of a collection of target genes that are significantly enriched in housekeeping genes functioning in ribosomes, translation, and energy. The synteny of open chromatin regions between different species may be due to the conservation of noncoding regions [9], indicating that *Aspergillus* species, which act as protein expression cell factories, exhibit the same potential functional elements in protein synthesis regulation.

The accessible chromatin regions identified by ATAC-seq are particularly valuable for the identification of *cis-*regulatory DNA elements [32, 48, 49]. By comparing the Tn5 digestion patterns of WT, TF-deleted strains, and naked genome, the strand-specific Tn5 integration patterns around TFBs were only observed in wild-type strain (Fig. 6a). In fact, TF does not exist in the naked genomic DNA and the TF-deleted strain, so the DNA footprints should not be enriched. Given the digestion bias of Tn5 transposase, these footprints are regarded as the pseudo footprints and several research proposed correction methods of TF footprinting [26, 42, 52]. However, there is no consensus on the significance of correcting DNase I or Tn5 transposase sequence bias. Li et al. [26] developed HINT-ATAC to measure the amount of the strand cleavage bias for distinct fragment sizes (All, Nfr, 1 N, and + 2 N) around distinct intervals near the TF binding site, and the results showed intricate strand-specific cleavage patterns relative to TF binding. Therefore, we reasoned that the strand-specific unbalanced pattern of Tn5 digestion on both sides of the footprints can be considered a true DNA footprint.

We systematically identified genome-wide DNA footprints from candidate accessible regions and performed de novo motif enrichment analysis on the footprints from *A. niger* CBS513.88 and SH2 (Fig. 5a, c). Clustering of TFs by family and analysis of *P* values indicated that the *A. niger* CBS513.88 and SH2 ATAC-seq footprints were enriched for de novo bZIP (motif GCTGAGTCAGCV) and basic helix-loop-helix (motif TCACGTGATC) families (Fig. 5c). The 775 genes predicted as DNA-binding TFs are primarily distributed among 37 classes of regulatory proteins, including 17 bZip family TFs and 10 bHLH family TFs in the *A. niger* genome [3, 5].

The bZip family of TFs that have been functionally analyzed in *A. niger* mainly include *hacA* (UPR) and *cpcA* (amino acid starvation). The function of other bZip TFs are associated with amino acid starvation (*jlbA*); oxidative stress (*napA*, *atfA*, and *atfB*); and sulfur metabolism (*cys*-3/*metR*), and they have mainly been characterized in homologous species, such as *A. nidulans* [53–55], *A. fumigatus* [14], and *A. oryzae* [56, 57]. The gene regulatory network of the bZIP family of *Neurospora crassa* [58] reveals that the bZIP family of TFs exerts consistent regulatory effects in response to environmental stresses, such as oxidative stress, amino acid starvation, and heavy metal pressure. Functional enrichment of the most proximal gene targeted by the bZip motif, GCTGAGTCAGCV, further demonstrates that this type of TF is associated with transcriptional regulation, which is the first step in regulating the stress response of filamentous fungi to the external environment.

The 10 TFs in the bHLH family, including homologous genes of *sclR*/*srbB*, *anbH1*, *srbA*, *palcA*, *INO4*, and *devR*, have not been well characterized in *A. niger*. However, in other species, the bHLH TFs have been postulated to be involved in hyphae growth and differentiation as well as specific metabolic pathways [36, 59]. The GO category of our E-box targeted genes found that they are involved in the biological process of translation, which is identical to the function of the bHLH family proteins that was predicted following *A. nidulans* genome sequence analysis [9]. Thus, we reasoned that bHLH TFs in *A. niger* have more unknown functions, and further characterization of bHLH TFs is urgently needed. The bHLH TFs recognize a consensus core element named the E-box (5′-CAVVTG-3′), and the G-box (5′-CACGTG-3′) is the most common form [59]; further, specific recognition of the sequence relies on flanking sequences [60, 61]. The E-box flanking sequences predicted herein contain not only the 5′ T residue but also the 3′ ATC residues. The 5′ T residue flanking the G-box determines major specificity by preventing the binding of other bHLH TFs, such as yeast PHO4 [61], whereas the significance of the 3′ residues is not fully known. The new motif CTGCCTGAGGCA is also a highly enriched motif according to the *P* value compared to all de novo previously unidentified TF binding motifs. GO functional analysis showed that this new motif CTGCCTGAGGCA drives the expression of target genes, and the GO molecular function categories include the cellular components of “ribosome” and “mitochondrial inner membrane;” the biological processes are related to “translation” and “metabolism.”

We constructed a minimal synthetic promoter suitable for filamentous fungi that was based on the yeast minimal promoter [62]. We used this promoter to validate the footprint library-driven UAS elements with TF binding site motifs, which provided a new method for in vivo validation of *A. niger* ATAC-seq-predicted binding sites containing TFBs in the absence of other omics data (DNase-seq, ChIP-seq). Our de novo motifs identified from enriched from ATAC-seq footprints were able to drive the expression of the targeted gene in vivo*.*

We further determined the enrichment of known TF binding motifs from ATAC-seq footprints by comparing ATAC-seq data from the *A. niger* wild-type strain and its TF-deleted strains. Footprints for these known TF binding motifs identified by ATAC-seq data were also verified as functional TFBs in vivo. Compared with *A. niger* CBS513.88, the enrichment *P* value of the TF motif in *A. niger* SH2 was significantly higher; the most differential values were observed for the CCAAT box, which represents a gene transcriptional activation domain [23], and the translation-related bHLH family [9]. This suggests that *A. niger* SH2 is a more suitable host for exogenous protein expression [4] at the TF level. The TF motif enrichment observed after growth in different carbon source conditions and in comparison to TF-deleted strains offer valuable information about TF dynamic responses in addition to the external environment and/or intrinsic regulatory factors. Transcriptome analysis of the known TFs *creA*, *amyR*, *pacC*, and *prtT* revealed that these transcription factors are involved in decentralized functions and do not cluster in categories directly related to TF function; this is even true for the pathway-specific transcription factor PrtT, which has a variety of functions that are not directly related to TF function that is clustered. Such results are ubiquitous in other studies using RNA-seq to study the function of transcription factors [13, 50, 63], which makes it particularly difficult to confirm the specific regulatory functions of transcription factors; as a result, the targeted GO term of a TF can only be determined by combining phenotypic changes [50] or ChIP-seq analysis [12, 13]. These functional clustering results are likely to be the effects of cascades of amplified changes caused by TF defects but are not the direct result of TF/DNA interaction. Through the co-regulation analysis of ATAC-seq and RNA-seq of SH2 TF-deleted strains (Fig. 7a), we effectively identified gene sets related to the corresponding TFs, and using GO clustering analysis, we screened genes and revealed that the obtained clustering results can accurately reflect the function of the experimentally verified TF (Fig. 7a) [35, 47, 64, 65]. Interestingly, we still predicted a different number of corresponding TF binding motifs in the TF knockout strains (delamyR, delprtT, delcreA, delpacC). This result may be caused by three factors: (i) None of these TFs are pioneers that change chromatin accessibility and the vacancies caused by TF defects are filled by other non-functional proteins. (ii) TF regulation is a complex dynamic process in vivo [8, 28], so that even if a TF was knocked out, there may be different TFs with alternate binding at the same DNA site. For example, the AmyR recognizing motif 5′-CGGN8(C/A)GG-3′ and the PrtT recognizing motif CCG(H)CGG compete for the binding sites in the same target gene during nitrogen and carbon source metabolism regulation [50]. (iii) These binding site identifications may be the result of a false positive. Unexpectedly, we discovered that CreA acts not only as repressors but also as activators, which was confirmed by footprint-driven reporter assays in vivo (Fig. 7c, d)*.* Our results contradict the fact that CreA is the repressor for CCR. It can only be speculated that CreA may play an unknown activation role in gene transcriptional regulation or that the predicted binding sites contain an activated binding motif for other unknown transcription factors.

## Conclusions

These data representing the ATAC-seq landscape of TF footprints will help in the exploration of genome-wide regulation of genes active in filamentous fungi, and the data will help to discover new regulatory factors and their potential sites of action. We demonstrated the usefulness of this resource by testing 25 noncoding DNA sites with predicted TF binding motifs driving reporter gene expression in the host strain. Future experiments will be required to validate the functional activity of more noncoding sites and investigate the role of specific TF footprints in controlling gene expression. The sites with *cis*-acting elements provide a large number of basic modules for artificially synthesized promoters, and our ATAC-seq data set will provide a rich resource for the identification of functional TFs in *A. niger*. In the future, ATAC-seq may be effectively applied to study other filamentous fungi whose genomes and transcriptomes are available but whose regulatory mechanisms have not yet been explored.

## Methods

### Strains and media

The specific genotypes of strains used in this study are shown in Additional file 11: Table S10. *E. coli* Mach 1 T1 (Invitrogen, America) was used for molecular cloning and was cultured in Luria-Bertani (LB) medium (1% NaCl, 1% tryptone, and 0.5% yeast extract) with 100 μg/mL ampicillin at 37 °C (both solid and liquid) while shaking at 200 rpm. Conidial *Aspergillus niger* CBS513.88 and *Aspergillus oryzae* niaD300 were cultured in PDA medium for spore germination, and they were cultured in DPY (also called YPD) medium (2% glucose, 1% peptone, 0.5% yeast extract, 0.5% KH_2_PO_4_ and 0.05% MgSO_4_·7H_2_O) for protoplast preparation. Aconidial *Aspergillus niger* SH2 (Δ*ku*Δ*pyrG*) was used as the host for the knockout of transcription factors and was cultured in modified liquid Czapek-Dox medium (CD) containing the following substances to support mycelial growth: 2% glucose, 0.3% NaNO_3_, 0.1% KH_2_PO_4_, 0.05% MgSO_4_·7H_2_O, 0.2% KCl, and 0.01% FeSO_4_·7H_2_O. When the OD reached 2.0, 1 ml of mycelia was inoculated into 100 ml of DPY medium and grown in a thermostatic shaker at 250 rpm and 30 °C for 40 h. Mycelia were harvested for protoplast preparation. To study the influence of carbon sources on the chromatin accessibility, the glucose in DPY was replaced with 2% maltose (MPY) [66] or strains were cultured in 2% fructose for 2 h, then transferred to CD with 0.1% fructose or CD with 1% glucose for CCR analysis [67]. *A. niger* HL-1 [68] was used as the expression host to produce the enzyme product of the glucose oxidase gene *goxC* (An01g14740). Solid CD (2% agarose) with 1% O-dianisidine (dissolved in methanol), 18% glucose, and 90 U/mL horseradish peroxidase was used to assess the color reaction following *goxC* expression.

### ATAC-seq library preparation

The *A. niger* strain CBS513.88, SH2 (including TF deletion strains and different culture conditions) and *A. oryzae* niaD300 (including *laeA* deletion and overexpression mutants) were grown to mid-log phase in DPY medium or conditional induction medium. Then, 1 g of mycelia per library was harvested using miracloth, and they were then rinsed with cold water and 0.8 M NaCl. Protoplasts were prepared by incubating mycelial samples in enzymatic lysis buffer composed of 0.8 M NaCl, 2% cellulase, 1% helicase, 1% lyticase, and 0.5% lysozyme, which were dissolved in the corresponding medium and were then filtered with miracloth and washed with cold STC buffer (10 mM Tri-HCl pH = 7.5, 1.2 M sorbitol, 50 mM CaCl_2_). Subsequently, 5 × 10^4^ protoplasts (cells) were incubated with 200 μl of lysis buffer (10 mM Tris-HCl, pH 7.4, 10 mM NaCl, 3 mM MgCl_2_, 0.05% (v/v) IGEPAL CA-630, 1 mM PMSF and 1xPIC (EDTA free)) for 10 min at 4 °C, washed with lysis buffer (without IGEPAL CA-630), and then incubated with 5 μl of Tn5 Transposase of TTE Mix V50 in 45 μL of 1× TTBL buffer (TruePrep® DNA Library Prep kit V2 for Illumina®, Vazyme, China) at 37 °C for 30 min. PCR was subsequently performed as previously described [25]. Libraries were purified with AMPure beads (Agencourt) to remove contaminating primer dimers. All libraries were sequenced with 50 bp paired-end reads on an Illumina Hiseq 2500 platform. For the control (*A. niger* CBS513.88 naked genome DNA), 5 ng of genomic DNA was used for the Tn5 transposase reaction. All the ATAC-seq data were submitted to the NCBI (Accession number, PRJNA566304, PRJNA587805 and PRJNA692847)

### Construction of transcription factor knockout and overexpression strains in *Aspergillus*

Different TFs, *creA* [47], *amyR* [65], *cpcA* [69], *pacC* [64], *prtT* [35], and *laeA* [37, 38], were searched on the *Aspergillus* genome database (www.aspgd.com), and homologous arms for gene knockout were designed based on the 1000-bp sequence flanking the CDS region of each gene, which were then amplified with primer sets listed in Additional file 12: Table S11. The uridine auxotrophic marker *pyrG* from *A. nidulans* was used as a screening marker for TF knockout. The upstream and downstream homologous arms of each TF obtained by PCR amplification were introduced along with the *pyrG* marker into a pMD20 T-Vector (TaKaRa, Japan) by NEBuilder (New England Biolabs, America), to obtain TF knockout vectors (pdelcreA, pdelamyR, pdelcpcA, pdelpacC, pdelprtT, and pdellaeA). The knockout cassettes that were linearized with *XbaI*, *EcoRI*, *ApaI*, *EcoRI*, *EcoRI*, and *BamHI* were introduced into *A. niger* SH2 and *A. oryzae* niaD300 by PEG-mediated protoplast transformation. In brief, protoplasts were prepared as the description in ATAC-seq library preparation. Protoplasts were resuspended in a certain amount of STC, and transformation system was made up of 100 μL linearized plasmid, 160 μL protoplasts, and 60 μL PEG. The mixture was incubated on the ice for 30 min, and then added another 1.5 mL PEG for 25 min at the room temperature. Finally, the mixture was pooled down on the hyperosmotic sucrose CD plate. After 5 days cultivated at 30 °C, transformants were obtained and identified. The cassettes for *laeA* overexpression digested with *ApaI* were transformed into *A. oryzae* niaD300 to yield the OElaeA strain. The TF knockout mutants in which homologous integration occurred were analyzed by PCR amplification for upstream and downstream localization, and they were further validated by qRT-PCR (Additional file 1: Figure S11). The primers used in this study are listed in Additional file 12: Table S11.

### Chromatin immunoprecipitation and ChIP-seq library preparations

To confirm the ATAC-seq data, we constructed PrtT-FLAG (CBS513.88 Δ*kusA*; *prtT*-3 × FLAG:*pyrG*) strain, in which the TF PrtT was drove by PglaA and labelled by 3 × FLAG tagging, for ChIP-seq analysis. Fungal conidia (10^6^/mL) were incubated in 100 mL DPY media at 30 °C under shaking at 200 rpm for 40 h. The mycelia was harvested, dried with two layers of miracloth, and transferred into buffer A for cross-linking. Cross-linking was done according to the protocol of [12]. Then samples were frozen in liquid nitrogen and lysed with Bullet Blender with the setting 12, 5 × 3 min with a 1-min ice interval in between. The lysed supernatant was collected for sonication (UCD 200 for 30 min) in ice water slurry with the program: 10 S on, 15 S off. Specific antibody anti-FLAG (Sigma, Anti-FLAG M2) was used for binding with the DNA/PrtT complex. For each standard IP, 50 μL chromatin, 2 μg antibody, and 450 μL lysis buffer were added and were kept on a magnetic stand. Precipitated sample was then washed and the immunoprecipitated DNA was digested with RNase A and purified with DNA extract kit (Qiagen, Cat. No. 27104). The extracted DNA was prepared using Illumina multiplex system and sequenced by Illumina HiSeq 2500 (Life Technologies).

### RNA extraction, qRT-PCR, and RNA-seq analysis

A small portion of the mycelium used to prepare the ATAC-seq library was left for RNA extraction. Total RNA was isolated using a HiPure Fungal RNA Kit (Magen, China). Reverse transcription was performed with a PrimeScript RT-PCR Kit (TaKaRa, Japan). Quantitative real-time PCR (qRT-PCR) was performed using an ABI 7500 Fast Real-Time PCR System to analyze TFs and the report gene *goxC*; the primers used are listed in Additional file 12: Table S11. The resulting libraries were sequenced at the Beijing Genomics Institute (BGI) with 50 bp single-end reads on the BGISEQ-500 (NCBI accession number, PRJNA588127). The RNA quality was assessed with an Agilent Bioanalyzer 2100 system to confirm that all samples had an RNA integrity value (above 7.0). All samples were prepared in duplicate. To obtain FASTQ format clean reads, all generated raw sequencing reads were filtered to remove reads with adaptors, reads in which unknown bases were greater than 10%, and low-quality reads. After filtering, clean reads were mapped to the genome and cDNA using HISAT [70] and Bowtie2 [71]. The gene expression level was quantified by RSEM [72], and the FPKM value of all samples is provided in Additional file 13: Supplementary Table S12. The DESeq2 method [73] was used to screen differentially expressed genes between the two groups.

### Mapping and normalization of ATAC-seq data

After removing adaptors using Trimmomatic [74], 50 bp paired-end ATAC-seq reads were mapped to the *Aspergillus* genome (Ensembl/ASM285 for *A. niger* and Ensemble/ASM18445 for *A. oryzae*) using Bowtie2 with parameters “--trim5 5 --trim3 15” and other default parameters. All reads aligned to the + strand and the − strand were offset by ± 5 bp, respectively, due to the Tn5 transposase introducing two cuts that were separated by 9 bp [75]. Mapped reads of SAM output were converted to a BAM format and sorted by Samtools [76]. Duplicate reads were removed using the default parameters of the Picard tools MarkDuplicates program (http://broadinstitute.github.io/picard/).

To visualize mapped reads, BAM format files were converted to bigwig format using the *bamCoverage* tool in deepTools2 with a bin size of 1 bp and with RPKM normalization. Heatmaps and average plots from the ATAC-seq data matrix were also generated using the “*computeMatrix*,” “*plotHeatmap*,” and “*plotProfile*” functions in the deepTools2 package [77]. Genome browser images were made using the integrative Genomics Viewer (IGV) [78] and bigwig files that were processed as described above. Fragments length between the pair-end adaptors were calculated with the program: Samtools view ATAC_sorted.bam | awk '$9>0' | cut -f 9 | sort | uniq -c | sort -b -k2, 2n | sed -e 's/^[ \t]*//' > fragment_length_count.txt and the desired length was extracted from SAM files by Python.

### Peak calling identified accessible chromatin regions

Accessible regions and peaks of each sample were identified using MACS2 [79] with the following parameters: -f BAMPE --nomodel --shift 100 --extsize 200. The narrowPeak files from the MACS2 output were used for further analysis. The *annotatePeaks.pl* tool of the HOMER program [45] was used with the default parameters to annotate the location of the identified peaks overlapping with genomic features, the TSS (transcription start site), the TTS (transcription termination site), exons, introns, and intergenic regions, all of which were annotated in the Ensemble *Aspergillus niger* CBS513.88 genome. Peaks that were 1 kb upstream of the TSS were associated with the nearest genes. These genes were then analyzed for overrepresented gene ontology and KEGG pathway using FungiFun 2.2.8 BETA (https://sbi.hki-jena.de/fungifun/) [80].

### Analysis of differential chromatin accessibility

To identify differentially mapped reads, we used Diffbind [81]. We used the processed ATAC-seq alignment bam file from Bowtie2 and the narrowPeak file from MACS2 for each sample. MA plots (log2-fold change vs. mean average) were used to visualize changes in chromatin accessibility for all peaks.

### Identification of footprints

Footprints were identified with pyDNase [44] using the default parameters. The Wellington footprinting algorithms [44] in pyDNase searched for TF footprints in areas of the genome that MACS2 identified as peaks. Wellington applied a beta-binomial distribution to estimate footprints and elevated the strand-specific activity of DNase or Tn5 transposase around transcription factor footprints among accessible chromatin regions.

### Design and synthesis of a minimal *Aspergillus* promoter for functional verification of CREs in vivo

With reference to the yeast minimal promoter screening system [62], we designed a minimal *Aspergillus* promoter for functional verification of CREs in vivo (Additional file 1: Figure S9a). The 67 bp 5′ UTR of the *Aspergillus nidulans TPI* gene [82] was used as a TSS. The TATA box and the TSS are separated by a 29-bp distance from the TSS in the *amyB* (An05g02100) promoter of *A. niger*, which facilitates successful loading of the transcription initiation complex, thereby initiating transcription by RNA polymerase. To ensure that the TATA box can be successfully opened during the transcription process, we manually added 8 AT-enriched sequences in front of the TA box to reduce the bond energy of the DNA [62]. These elements were linked together by fusion PCR amplification to form a core promoter region (Additional file 1: Figure S9a). The glucose oxidase *goxC* (An01g14740) was used as the reporter gene, which could catalyze the oxidation of D-glucose at carbon 1 into D-glucono-1, 5-lactone and hydrogen peroxide. Then the peroxidase catalyzes the decomposition of hydrogen peroxide while oxidizing O-anisidine, thus resulting in a red phenotype [83] (Additional file 1: Figure S9b). To further refine the entire reporting system, the selection marker *pyrG* was integrated before the footprints to separate the footprints from other genomic sequences, ensuring that transcription of the core sequence was not affected by other sequences. The footprints contained a transcription factor binding site (TFBS) that helped stabilize RNAP to enhance transcription efficiency. By constantly changing footprints and monitoring the transcription of downstream reporter genes, it was possible to identify whether the footprints containing the TFBs function in vivo. The 1 kb flanking sequence of the glucoamylase gene (*glaA*) acts as a homologous arm, and the aim was that the sequence successfully integrated into the glucoamylase gene locus, ensuring that all open reading frames were at the same position in the genome [84]. All the report plasmids were linearized with *XbaI* and transformed into *A. niger* by PEG-mediated protoplast transformation.

### Discovery of de novo transcription factor motifs

ATAC-seq footprints that were identified from each sample were used to discover de novo transcription factor motifs. The boundaries of the start and stop coordinates of ATAC-seq footprints were lengthened by 10 bp upstream and downstream. We then used the *findMotifsGenome.pl* tool of the HOMER program to identify overrepresented transcription factor sites (transcription factor motifs). Using the *annotatePeaks.pl* tool of HOMER with the –m parameter, HOMER de novo motifs were then used to search the original set of sequences to identify all instances of motifs.

### Analysis of known motif enrichment within footprints

The known motif files in the HOMER format were created manually with the *seq2profile.pl* tool of HOMER. The known motifs were then used to search the footprints of each sample to identify all instances of motifs using the *annotatePeaks.pl* tool of HOMER with the –m parameter.

## Supplementary Information

**Additional file 1: Figure S1**. Fragment length distribution of Tn5 transposase integration. **Figure S2**. qRT-PCR analysis of *A. niger* cultured in different condition. **Figure S3**. Enrichment of PrtT binding motif from the ChIP-seq data. **Figure S4**. Visually display of the PrtT binding site. **Figure S5**. Clustering analysis of ATAC-seq peaks for *Aspergillus oryzae* niaD300 and its *laeA* mutants. **Figure S6**. Comparison of ATAC-seq peaks and RNA-seq signal among *A.oryzae* niaD300 and *laeA* mutant. **Figure S7**. *De novo* motifs of SH2 TF-deficient strains. **Figure S8**. GO analysis of 3 *de novo* predicted over-represented TFBs targeting genes. **Figure S9**. The minimal promoter structure and the verification of its function. **Figure S10**. *In vivo* functional verification of AreA targeting sites. **Figure S11**. qRT-PCR verification of TF knockout strains of *A.niger* SH2 and *A.oryzae* niaD300.

**Additional file 2: Supplementary Table S1**. Sequencing data quality and mapping rate analysis of the ATAC-seq samples.

**Additional file 3: Supplementary Table S2**. Chromatin accessible regions (Peaks) of each sample identified by MACS2.

**Additional file 4: Supplementary Table S3**. Syntenic orthologues of ATAC-seq peaks identified between *A. niger* CBS513.88 and *A. oryzae* niaD300.

**Additional file 5: Supplementary Table S4**. Differential peaks analysis of *A. niger* and its mutants.

**Additional file 6: Supplementary Table S5**. Differential peaks and SM cluster analysis of *A. oryzae* and its *laeA* mutants.

**Additional file 7: Supplementary Table S6**. DNA footprints of *A. niger* wild-type strains and TF-deleted strains.

**Additional file 8: Supplementary Table S7**. *De novo* TFBs of *A. niger* wild-type strains and TF-deleted strains with a *P* value threshold of 1E-50 by HOMER.

**Additional file 9: Supplementary Table S8**. The physical position and gene function of DNA instances containing the TFBs mapped to the *A. niger* genome.

**Additional file 10: Supplementary Table S9**. Motif instances of known TFs from footprints by comparing *A. niger* WT strains and the TF-deleted strains.

**Additional file 11: Supplementary Table S10**. Genotype of strains used in this study.

**Additional file 12: Supplementary Table S11**. Primers used in this study

**Additional file 13: Supplementary Table S12**. FPKM value of all the *A. niger* RNA-seq samples.

**Additional file 14: Supplementary Table S13**. Original data of all the figures and additional files.

## Data Availability

All data generated or analyzed during this study are included in this published article, its supplementary information files, and publicly available repositories. The raw ATAC-seq and ChIP-seq sequencing data have been deposited in the NCBI SRA database under the accession number PRJNA566304, PRJNA587805, PRJNA692847, and PRJNA692789. The raw RNA-seq sequencing data have been deposited in the NCBI SRA database under the accession number PRJNA588127. The Supporting data are included in the additional files 2, 3, 4, 5, 6, 7, 8, 9, 10, 11, 12 and 13. A file providing individual data values was submitted as Additional file 14.

## Abbreviations

ATAC-seq: Assay for transposase-accessible chromatin using sequencing
TF: Transcription factor
TFBs: Transcription factor binding sites
ChIP-seq: Chromatin immunoprecipitation sequencing
CREs: *cis*-regulatory elements
TSS: Transcription start site
TTS: Transcription terminator site
SMs: Secondary metabolites
PKSs: Polyketide synthases
NRPSs: Non-ribosomal peptide synthetases
AF: Aflatoxin
DEGs: Differential expression genes
OC: Open chromatin
CCR: Carbon catabolite repression
