# Supplementary material for: Profiling of chromatin accessibility identifies transcription factor binding sites across the genome of Aspergillus species
Source: BMC Biol. 2021 Sep 6;19:189. doi: 10.1186/s12915-021-01114-0 (PMC8419926; doi:10.1186/s12915-021-01114-0)
Supplement: Supplementary file 1 — Additional file 1: Figure S1. Fragment length distribution of Tn5 transposase integration. Figure S2. qRT-PCR analysis of A. niger cultured in different condition. Figure S3. Enrichment of PrtT binding motif from the ChIP-seq data. Figure S4. Visually display of the PrtT binding site. Figure S5. Clustering analysis of ATAC-seq peaks for Aspergillus oryzae niaD300 and its laeA mutants. Figure S6. Comparison of ATAC-seq peaks and RNA-seq signal among A.oryzae niaD300 and laeA mutant. Figure S7. De novo motifs of SH2 TF-deficient strains. Figure S8. GO analysis of 3 de novo predicted over-represented TFBs targeting genes. Figure S9. The minimal promoter structure and the verification of its function. Figure S10. In vivo functional verification of AreA targeting sites. Figure S11. qRT-PCR verification of TF knockout strains of A.niger SH2 and A.oryzae niaD300. [file 12915_2021_1114_MOESM1_ESM.pdf]

# Profiling of chromatin accessibility identifies transcription factor binding sites across the genome of *Aspergillus* species

Lianggang Huang[1], Xuejie Li[1], Liangbo Dong[1], Bin Wang[1,2,*], Li Pan[1,2,*]

[1] School of Biology and Biological Engineering, South China University of Technology, Guangzhou Higher Education Mega Center, Guangzhou, 510006, China

[2] Guangdong Provincial Key Laboratory of Fermentation and Enzyme Engineering, South China University of Technology, Guangzhou Higher Education Mega Center, Guangzhou, 510006, China

* To whom correspondence should be addressed. Tel:+86-20-39380601; Fax: +86-20-39380698; Email: btlipan@scut.edu.cn; btbinwang@scut.edu.cn

## Supplementary Figures

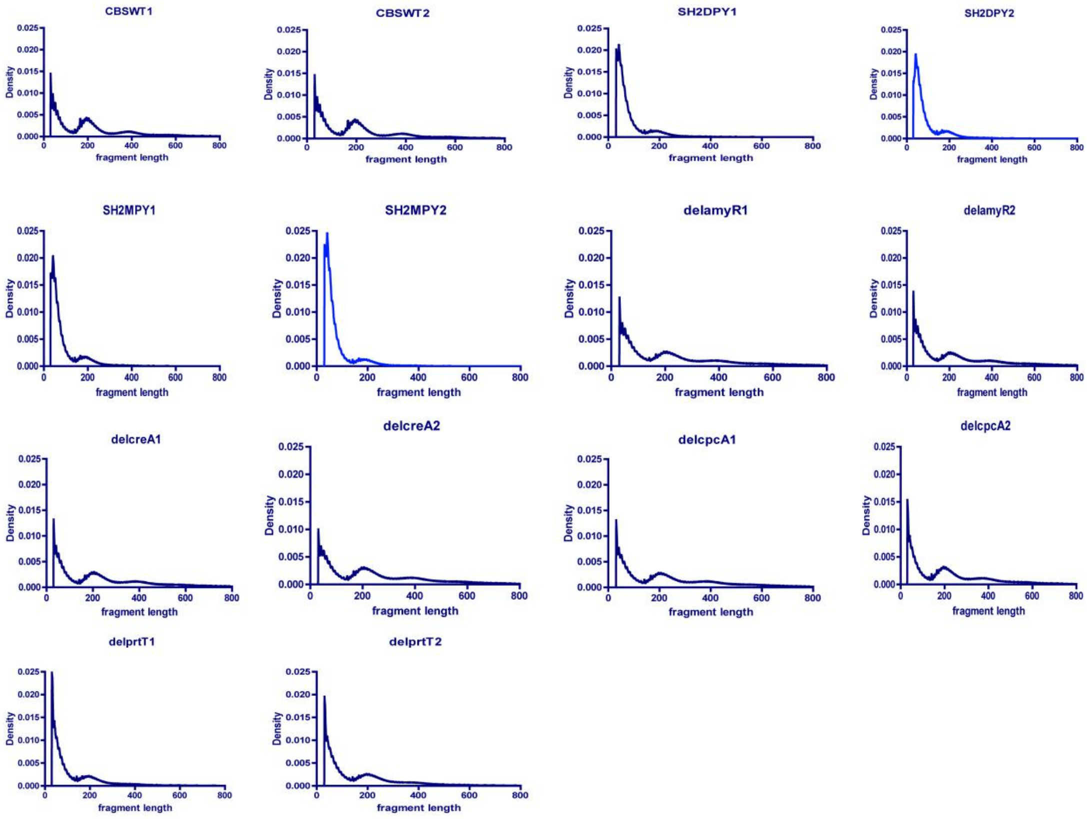


**Supplementary Figure S1** Fragment length distribution of Tn5 transposase integration in all samples.

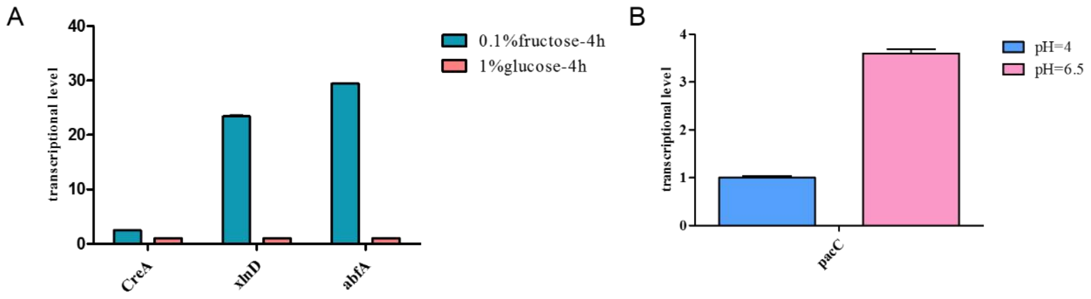


**Supplementary Figure S2** qRT-PCR analysis of *A. niger* cultured in different condition. (A) Analysis of *creA* and its target genes *xlnD*, *abfA* in carbon source inducing. (B) Analysis of *pacC* in pH=4.0 and pH=6.5.

| Rank | Motif | Name | P-value | log P-pvalue | q-value (Benjamini) | # Target Sequences with Motif | % of Targets Sequences with Motif | # Background Sequences with Motif | % of Background Sequences with Motif | Motif File | SVG |
|---|---|---|---|---|---|---|---|---|---|---|---|
| 1 | 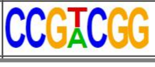 | Zn2Cys6_PrtT | 1e-18 | -4.149e+01 | 0.0000 | 35.0 | 20.59% | 1542.0 | 3.14% | motif file (matrix) | svg |

**Supplementary Figure S3** Enrichment of PrtT binding motif from the ChIP-seq data.

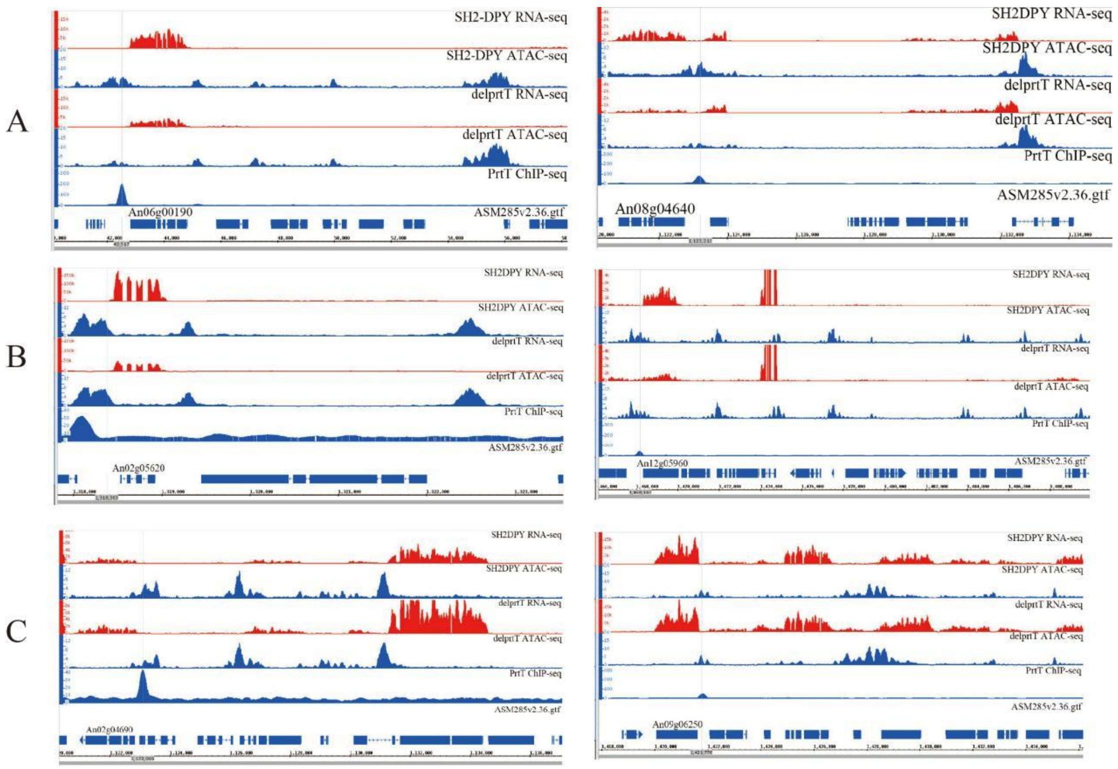


**Supplementary Figure S4** Visually display of the coupling analysis of ATAC-seq, ChIP-seq and RNA-seq using IGV browser. The grey vertical line indicates the PrtT binding site.

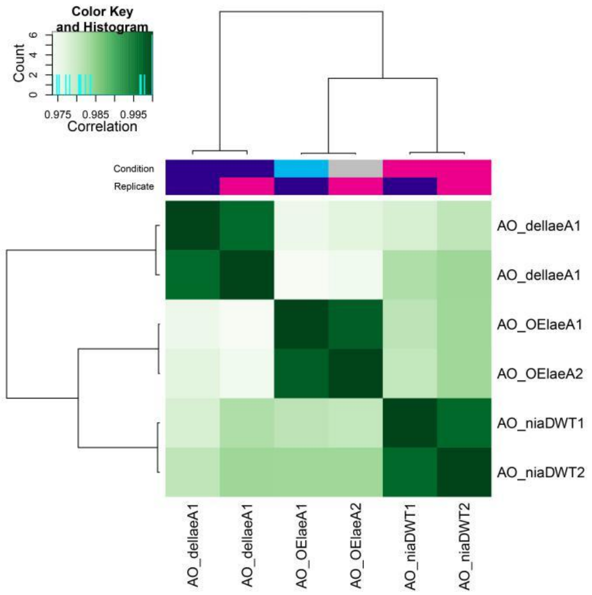


**Supplementary Figure S5** Clustering analysis of ATAC-seq peaks for *Aspergillus oryzae* niaD300 and its *laeA* mutants. 1 and 2 represents the independent replicate.

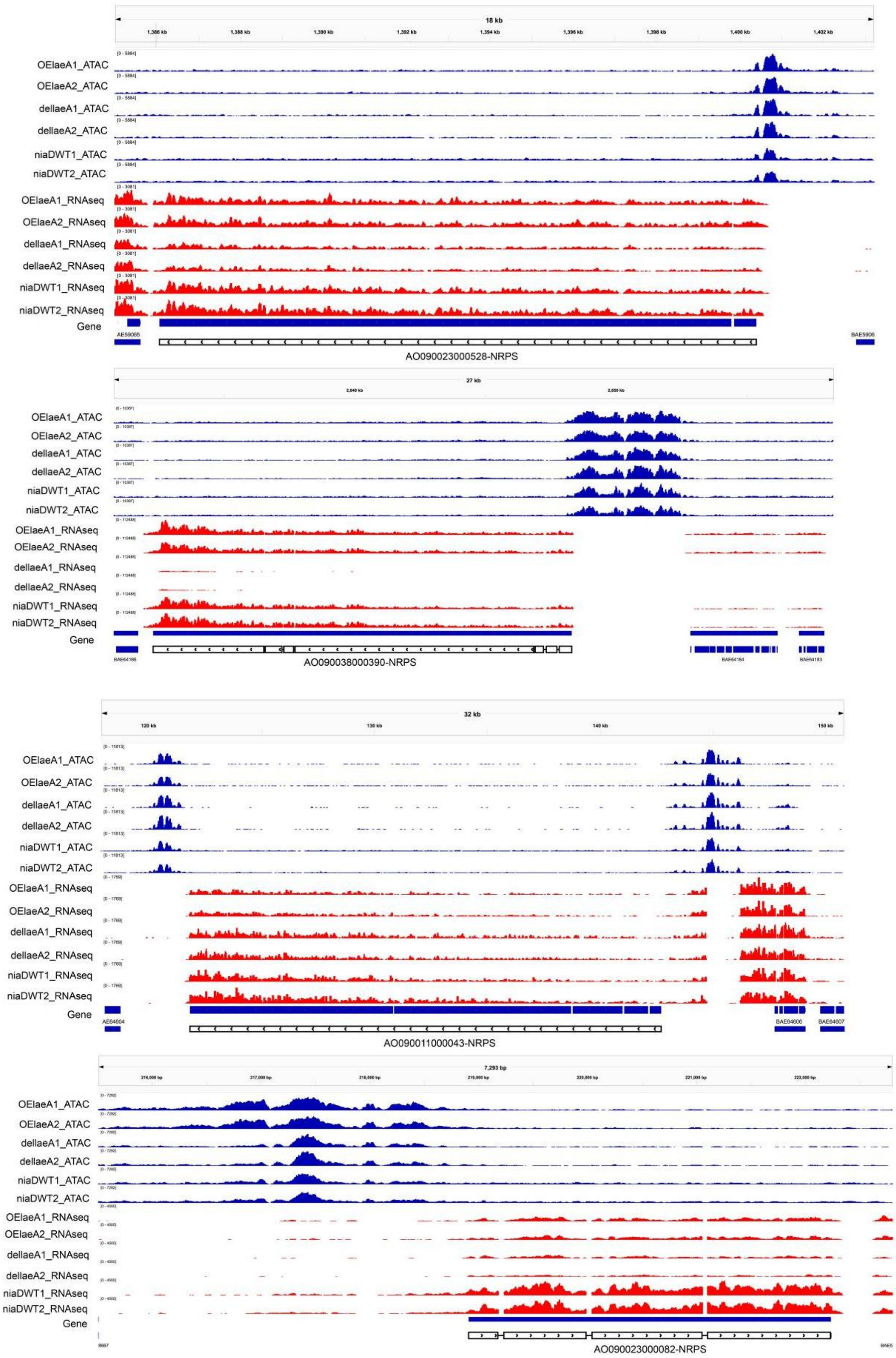

18 kb
OElaeA1_ATAC
OElaeA2_ATAC
dellaeA1_ATAC
dellaeA2_ATAC
niaDWT1_ATAC
niaDWT2_ATAC
OElaeA1_RNAseq
OElaeA2_RNAseq
dellaeA1_RNAseq
dellaeA2_RNAseq
niaDWT1_RNAseq
niaDWT2_RNAseq
Gene
AO090023000528-NRPS
27 kb
OElaeA1_ATAC
OElaeA2_ATAC
dellaeA1_ATAC
dellaeA2_ATAC
niaDWT1_ATAC
niaDWT2_ATAC
OElaeA1_RNAseq
OElaeA2_RNAseq
dellaeA1_RNAseq
dellaeA2_RNAseq
niaDWT1_RNAseq
niaDWT2_RNAseq
Gene
AO090038000390-NRPS
32 kb
120 kb
130 kb
140 kb
150 kb
OElaeA1_ATAC
OElaeA2_ATAC
dellaeA1_ATAC
dellaeA2_ATAC
niaDWT1_ATAC
niaDWT2_ATAC
OElaeA1_RNAseq
OElaeA2_RNAseq
dellaeA1_RNAseq
dellaeA2_RNAseq
niaDWT1_RNAseq
niaDWT2_RNAseq
Gene
AO090011000043-NRPS
7,293 bp
OElaeA1_ATAC
OElaeA2_ATAC
dellaeA1_ATAC
dellaeA2_ATAC
niaDWT1_ATAC
niaDWT2_ATAC
OElaeA1_RNAseq
OElaeA2_RNAseq
dellaeA1_RNAseq
dellaeA2_RNAseq
niaDWT1_RNAseq
niaDWT2_RNAseq
Gene
AO090023000082-NRPS

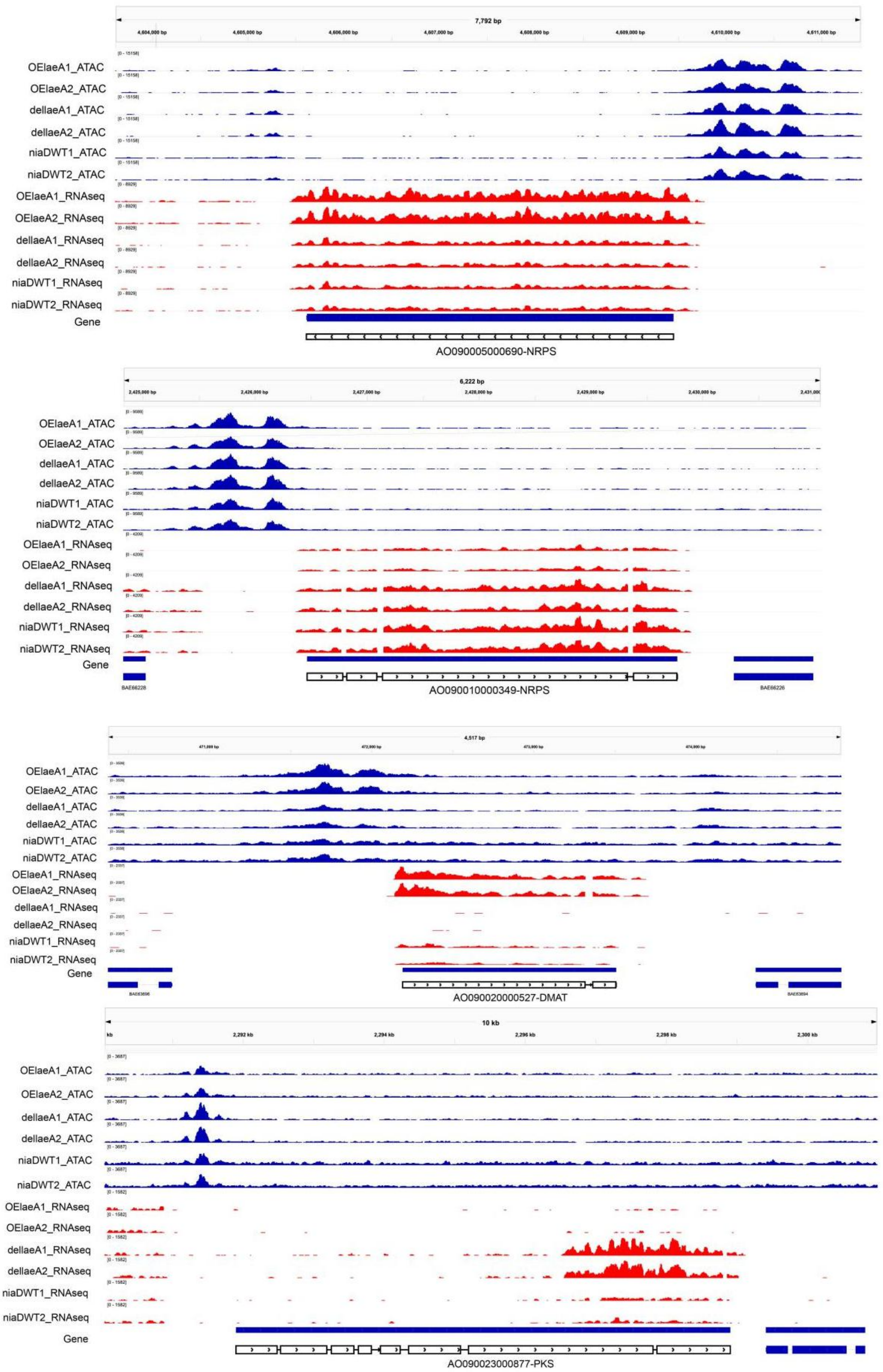


**Supplementary Figure S6** Comparison of ATAC-seq peaks and RNA-seq signal among *A.oryzae* niaD300 wild type strain, dellaeA mutant, and OElaeA mutant. The representative secondary metabolic biosynthesis genes (including 6 NRPSs, 1 DMAT and 1 PKS) are shown by IGV browser. The red peaks represent for ATAC-seq signal

and blue for RNA-seq signal.

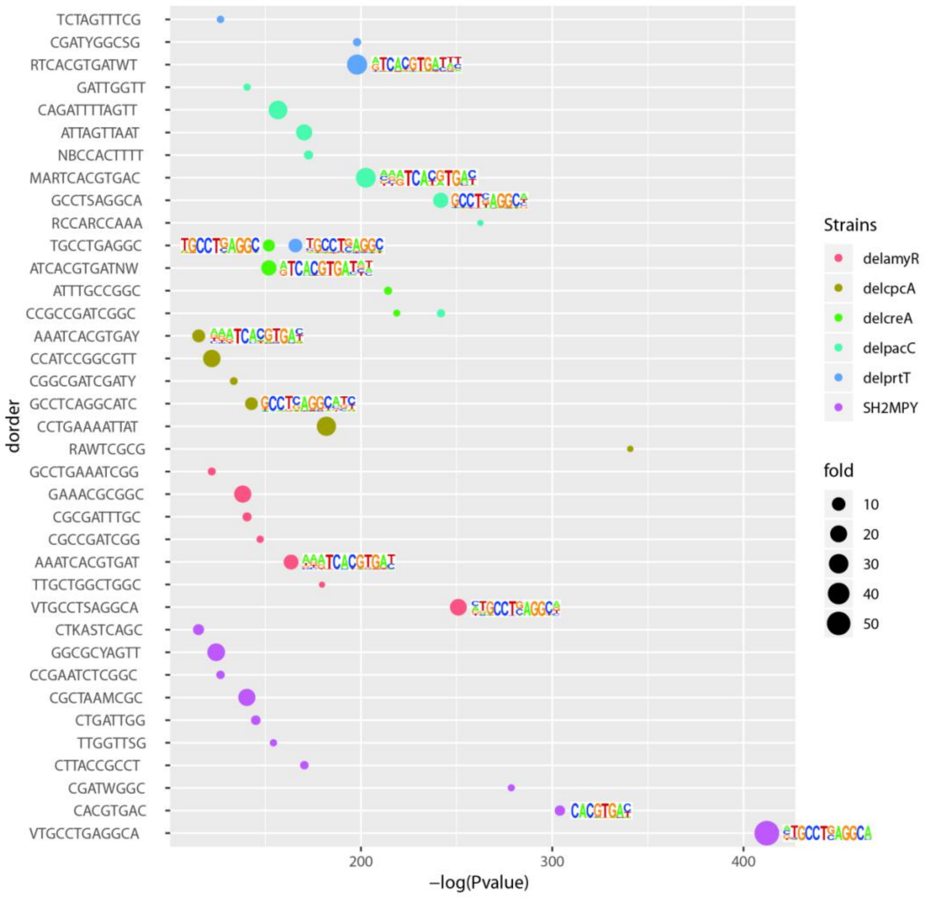


**Supplementary Figure S7** *De novo* motifs of SH2 TF-deficient strains with P-value threshold of 1E-50. The circle size represents the enrichment fold. The top three transcription factor motifs in *A. niger* SH2 TF deletion mutants are labeled with the corresponding binding motifs. The x-axis represents the -log(Pvalue).

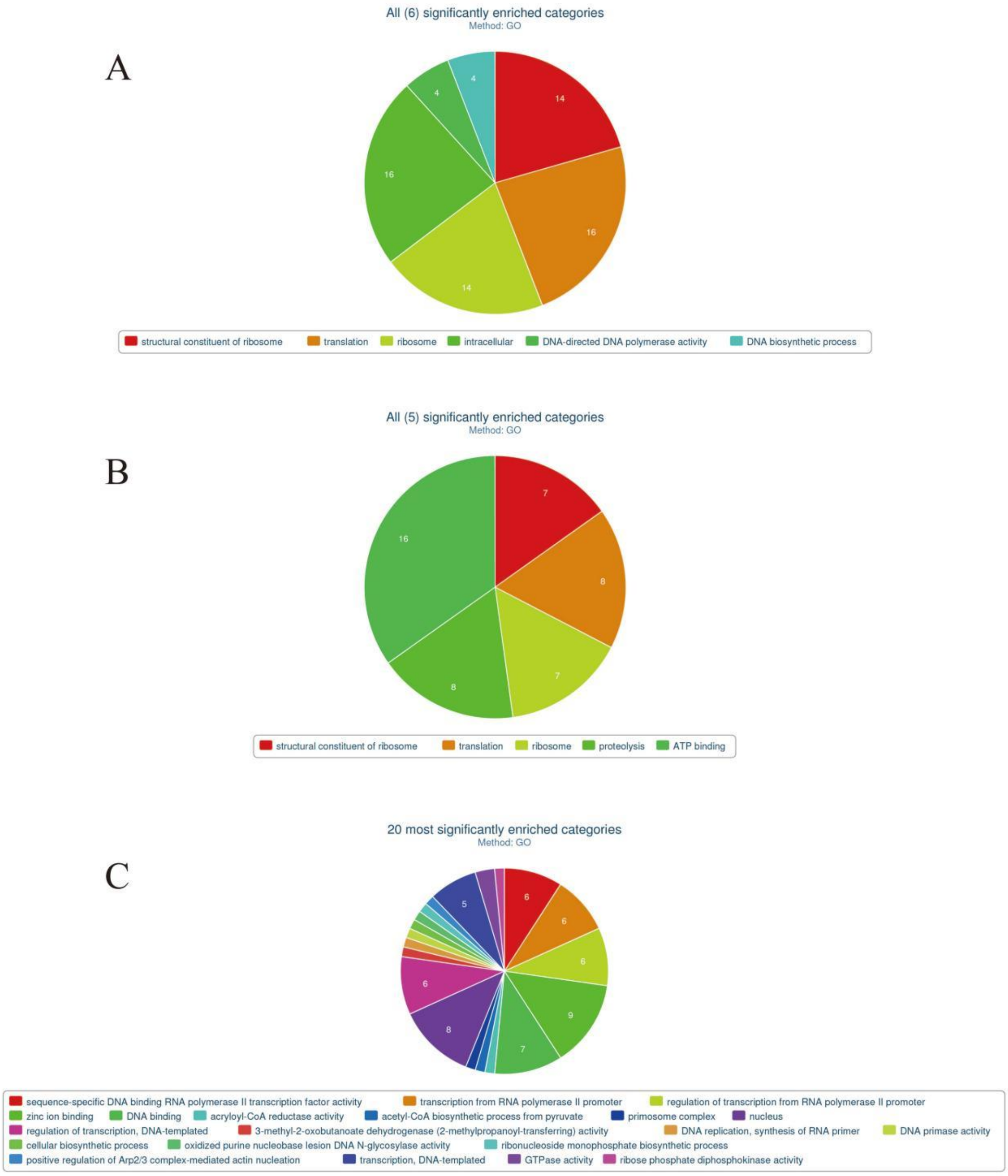


**Supplementary Figure S8** GO analysis of 3 *de novo* predicted over-represented TF-binding motif targeting genes. (A) GO category of **269** genes under the control of motif M2:TCACGTGATC; (B) GO category of **154** genes under the control of motif M3:CTGCCTGAGGCA; (C) GO category of **54** genes under the control of motif M5:GCTGAGTCAGCV.

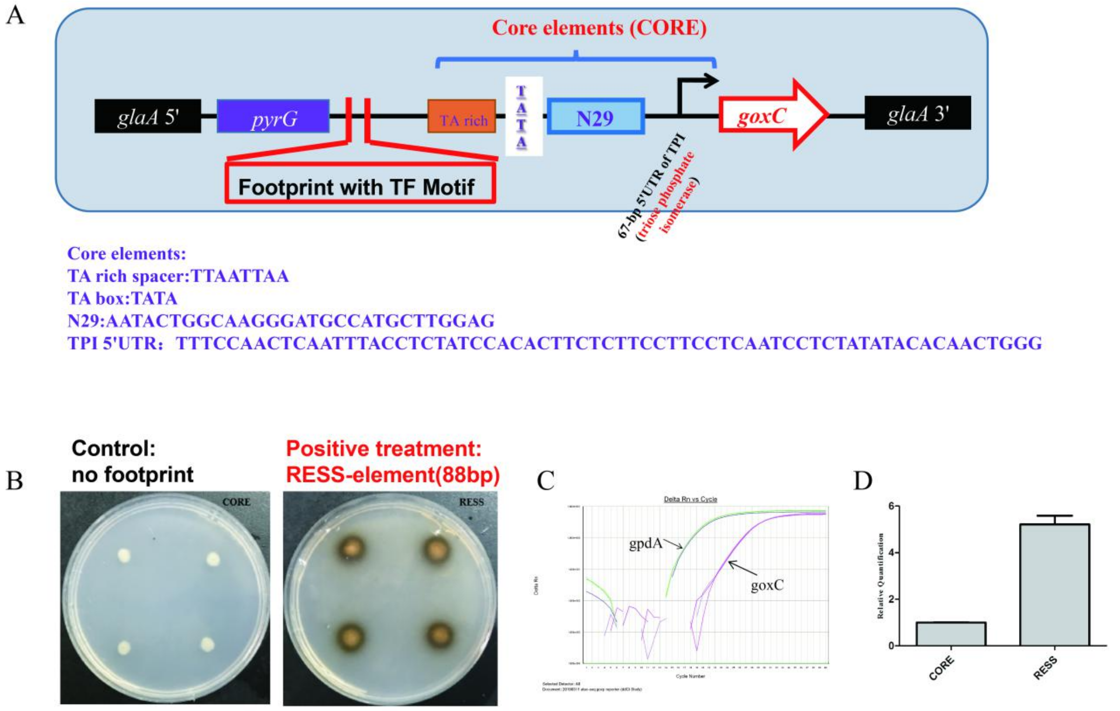


**Supplementary Figure S9** The minimal promoter structure and the verification of its function. (A) The structure of minimal core element promoter. 5' and 3' sequences of *glaA* gene served as homologous arms to ensure the integration of the expression cassette into the same position in *A. niger* genome through homologous recombination. (B) The minimal promoter-driven reporting system was validated *in vivo* using the RESS sequence. Control: no footprint (left); Positive treatment: RESS element served as the footprint with TF motif (right); (C) Fluorescence quantitative plot of the control strain (CORE). The *gpdA* gene was served as the reference gene. (D) qRT-PCR analysis of *goxC* driven by CORE and RESS.

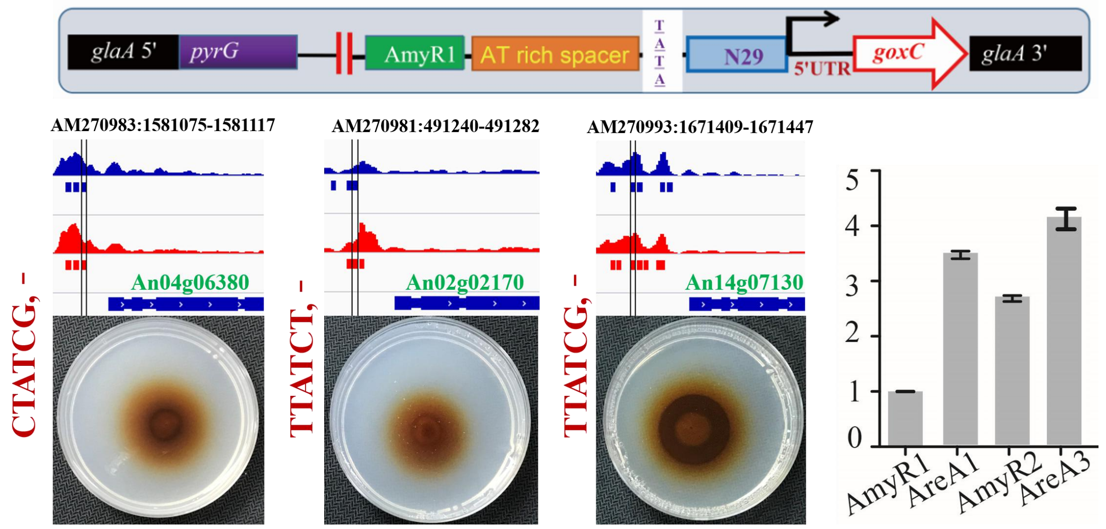


**Supplementary Figure S10** *In vivo* functional verification of AreA targeting sites. The red dividing line represents the location of the footprints to be verified. The Y-axis indicates the binding motif in the footprint instances. The driving strength of the AreA footprint was detected by qRT-PCR. AmyR1 served as the control.

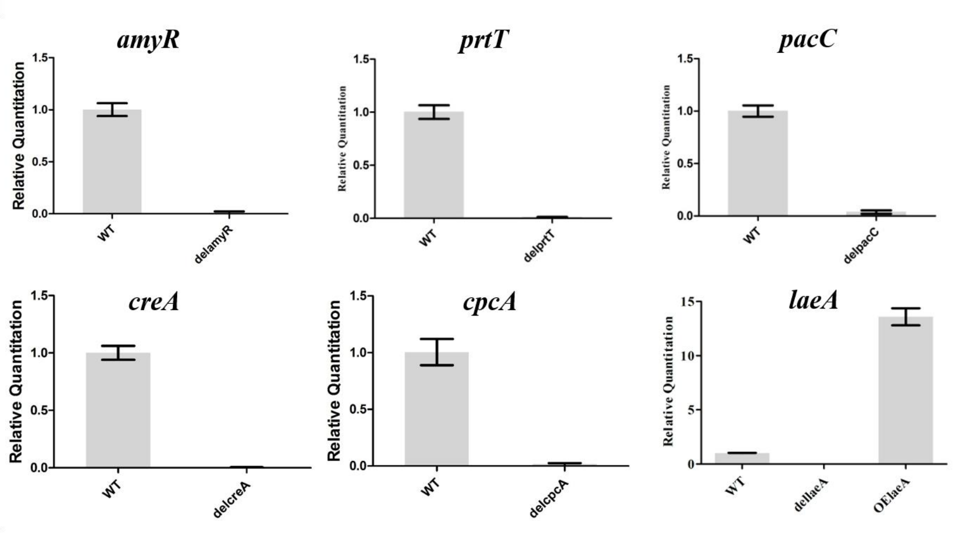


**Supplementary Figure S11** qRT-PCR verification of TF knockout strains of *A.niger* SH2 and *A.oryzae* niaD300. The expression of target genes were normalized to the expression level of the endogenous control gene *gpdA*.
