# Supplementary material for: Profiling of chromatin accessibility identifies transcription factor binding sites across the genome of Aspergillus species
Source: BMC Biol. 2021 Sep 6;19:189. doi: 10.1186/s12915-021-01114-0 (PMC8419926; doi:10.1186/s12915-021-01114-0)
Supplement: Supplementary file 11 — Additional file 11: Supplementary Table S10. Genotype of strains used in this study. [file 12915_2021_1114_MOESM11_ESM.docx]

**Supplementary Table S10 Genotype of strains used in this study**

| Strain | Genotype | Source |
| --- | --- | --- |
| *[E. coli](https://www.sciencedirect.com/topics/biochemistry-genetics-and-molecular-biology/escherichia-coli)* Mach 1T1 | Host for molecular clone | Invitrogen |
| *A.niger* CBS513.88 | Wild type | DSM |
| *PrtT-3×FLAG* | prtT-3×FLAG:pyrG | CBS513.88∆*ku*∆*pyrG* |
| *A.oryzae* RIB40 | Wild type | NBRC |
| *A.oryzae* niaD300 | ∆*niaD* | *A.oryzae* RIB40 |
| *A.oryzae* ∆*ku*∆*pyrG* | ∆*pyrG*∆*ku*::*ptrA* | *A.oryzae* niaD300 |
| *A.oryzae* ∆*ku*∆*pyrG dellaeA* | ∆*niaD*∆*laeA*::*pyrG* | This study |
| *A.oryzae* ∆*ku*∆*pyrG OElaeA* | ∆*niaD*,PglaA-*laeA*:*pyrG* | This study |
| *A.niger* SH2 | Wild type | Enzyme fermentation factory |
| *A. niger* HL-1 | Wild type | Enzyme fermentation factory |
| *A.niger* SH2∆*ku*∆*pyrG* | ∆*ku*∆*pyrG* | This study |
| *A.niger* SH2∆*creA* | ∆*ku*∆*pyrG*∆*creA*::*pyrG* | This study |
| *A.niger* SH2∆*amyR* | ∆*ku*∆*pyrG*∆*amyR*::*pyrG* | This study |
| *A.niger* SH2∆*cpcA* | ∆*ku*∆*pyrG*∆*cpcA*::*pyrG* | This study |
| *A.niger* SH2∆*prtT* | ∆*ku*∆*pyrG*∆*prtT*::*pyrG* | This study |
| *A.niger* SH2∆*pacC* | ∆*ku*∆*pyrG*∆*pacC*::*pyrG* | This study |
| *A. niger* HL-1∆*ku*∆*pyrG* | ∆*ku*∆*pyrG* | This study |
| *A. niger* HL-1CORE | ∆*ku*∆*pyrG*CORE-*goxC*:*pyrG* | This study |
| *A. niger* HL-1RESS | ∆*ku*∆*pyrG*RESSCORE-*goxC*:*pyrG* | This study |
| *A. niger* HL-1bHLH1 | ∆*ku*∆*pyrG*bHLH1CORE-*goxC*:*pyrG* | This study |
| *A. niger* HL-1bHLH2 | ∆*ku*∆*pyrG*bHLH2CORE-*goxC*:*pyrG* | This study |
| *A. niger* HL-1bZIP1 | ∆*ku*∆*pyrG*bZIP1CORE-*goxC*:*pyrG* | This study |
| *A. niger* HL-1bZIP2 | ∆*ku*∆*pyrG*bZIP2CORE-*goxC*:*pyrG* | This study |
| *A. niger* HL-1bZIP3 | ∆*ku*∆*pyrG*bZIP3CORE-*goxC*:*pyrG* | This study |
| *A. niger* HL-1AGGCA1 | ∆*ku*∆*pyrG* AGGCA1CORE-*goxC*:*pyrG* | This study |
| *A. niger* HL-1AGGCA2 | ∆*ku*∆*pyrG* AGGCA2CORE-*goxC*:*pyrG* | This study |
| *A. niger* HL-1AGGCA3 | ∆*ku*∆*pyrG* AGGCA3CORE-*goxC*:*pyrG* | This study |
| *A. niger* HL-1amyR1 | ∆*ku*∆*pyrG* amyR1CORE-*goxC*:*pyrG* | This study |
| *A. niger* HL-1amyR2 | ∆*ku*∆*pyrG* amyR2CORE-*goxC*:*pyrG* | This study |
| *A. niger* HL-1amyR3 | ∆*ku*∆*pyrG* amyR3CORE-*goxC*:*pyrG* | This study |
| *A. niger* HL-1amyR4 | ∆*ku*∆*pyrG* amyR4CORE-*goxC*:*pyrG* | This study |
| *A. niger* HL-1prtT1 | ∆*ku*∆*pyrG* prtT1CORE-*goxC*:*pyrG* | This study |
| *A. niger* HL-1prtT2 | ∆*ku*∆*pyrG* prtT2CORE-*goxC*:*pyrG* | This study |
| *A. niger* HL-1prtT3 | ∆*ku*∆*pyrG* prtT3CORE-*goxC*:*pyrG* | This study |
| *A. niger* HL-1CBC1 | ∆*ku*∆*pyrG* CBC1CORE-*goxC*:*pyrG* | This study |
| *A. niger* HL-1CBC2 | ∆*ku*∆*pyrG* CBC2CORE-*goxC*:*pyrG* | This study |
| *A. niger* HL-1CBC3 | ∆*ku*∆*pyrG* CBC3CORE-*goxC*:*pyrG* | This study |
| *A. niger* HL-1creA1 | ∆*ku*∆*pyrG* creA1-amyR1CORE-*goxC*:*pyrG* | This study |
| *A. niger* HL-1creA2 | ∆*ku*∆*pyrG* creA2-amyR1CORE-*goxC*:*pyrG* | This study |
| *A. niger* HL-1creA3 | ∆*ku*∆*pyrG* creA3-amyR1CORE-*goxC*:*pyrG* | This study |
| *A. niger* HL-1creA4 | ∆*ku*∆*pyrG* creA4-amyR1CORE-*goxC*:*pyrG* | This study |
| *A. niger* HL-1areA1 | ∆*ku*∆*pyrG* areA1-amyR1CORE-*goxC*:*pyrG* | This study |
| *A. niger* HL-1areA2 | ∆*ku*∆*pyrG* areA2-amyR1CORE-*goxC*:*pyrG* | This study |
| *A. niger* HL-1areA3 | ∆*ku*∆*pyrG* areA3-amyR1CORE-*goxC*:*pyrG* | This study |
