# Supplementary material for: Profiling of chromatin accessibility identifies transcription factor binding sites across the genome of Aspergillus species
Source: BMC Biol. 2021 Sep 6;19:189. doi: 10.1186/s12915-021-01114-0 (PMC8419926; doi:10.1186/s12915-021-01114-0)
Supplement: Supplementary file 12 — Additional file 12: Supplementary Table S11. Primers used in this study [file 12915_2021_1114_MOESM12_ESM.docx]

**Supplementary Table S11** Primers used in this study

| Name | Primer Sequences(5'→3') | |
| --- | --- | --- |
| **Primers used for TFs knock-out** | | |
| ΔamyR-up-F | | ATCTACTAGTCATATGGATTGAATTCCTGGTTTGTAAAGCAGGTGGCAC |
| ΔamyR-up-R | | CGCGTTCTCGAGGAAGTTGCTTGTATGCGGAGACAAGTGTGAC |
| ΔamyR-down-F | | AACGGTATTGACTAAAAGGGGAAGGCAACTACGACGATGACG |
| ΔamyR-down-R | | TCGGTACCCGGGGATCCGATATTCATGTCTCCTGCGGAAATGG |
| ΔcpcA-up-F | | ATCTACTAGTCATATGGATTGGGCCCGGTGATTGGCGGCGAGATCCG |
| ΔcpcA-up-R | | CGCGTTCTCGAGGAAGTTGCGATGCGGCGATGCTTCAGATATG |
| ΔcpcA-down-F | | AACGGTATTGACTAAAAGGGTCTAAAAAGTTTCTTGGGTTTTC |
| ΔcpcA-down-R | | TCGGTACCCGGGGATCCGATGGGCCCCATCTAGGGGACTCCCTGC |
| ΔpacC-up-F | | ATCTACTAGTCATATGGATTGAATTCAATGCCATGATGGGGGATGACT |
| ΔpacC-up-R | | CGCGTTCTCGAGGAAGTTGCTGCCAAGAAAAATCTTTCTTGTCC |
| ΔpacC-down-F | | AACGGTATTGACTAAAAGGGGTTGTTTGCTGGTCACTATCTTTG |
| ΔpacC-down-R | | TCGGTACCCGGGGATCCGATCTCCTTCTTTCTCTCCTCTTTTC |
| ΔcreA-up-F | | ATCTACTAGTCATATGGATTGTGTGTAAGAGCGGTAGATACCC |
| ΔcreA-up-R | | CGCGTTCTCGAGGAAGTTGCGTGAAGCTTGTCCCAAGACCGA |
| ΔcreA-down-F | | AACGGTATTGACTAAAAGGGTTCGAACATTCTTCAGCCACACG |
| ΔcreA-down-R | | TCGGTACCCGGGGATCCGATTCTAGAAGAGATAACGTGAACCCGGCATT |
| ΔprtT-up-F | | CTACTAGTCATATGGATTGAATTCAGAGAGAAAATTTTTCTCTCCTTGG |
| ΔprtT-up-R | | CGCGTTCTCGAGGAAGTTGCGTCATCAAACAAAGTCCACTTTGG |
| ΔprtT-down-F | | AACGGTATTGACTAAAAGGGGTAATCGAGATCGGGTTGGAAAG |
| ΔprtT-down-R | | GTACCCGGGGATCCGATTTCCTTCCAAAAGTTACACCTACAT |
| ΔlaeA-up-F | | ggatccTACGACGATGATTGTAGAAGGGAC |
| ΔlaeA-up-R | | CTGTCTTTGGCAAGCGGGAAATTGGTAGTGGTGGACGAGGG |
| ΔlaeA-redown-F | | TTCCCGCTTGCCAAAGACAG |
| ΔlaeA-redown-R | | GGCGCGTTCTCGAGGAAGTTGCCGGCTTGACCAGGAAGACGA |
| ΔlaeA-down-F | | ATGTGTAACGGTATTGACTAAAAGGGTAACCTGGCTGTGGCTCCTTC |
| ΔlaeA-down-R | | ggatccATTCTTCCCTCCGCTCCATTTC |
| pyrG-F | | GCAACTTCCTCGAGAACGCGC |
| pyrG-R | | CCCTTTTAGTCAATACCGTTACAC |
| **Primers used for laeA overexpression** | | |
| PamyB-F | | CTGCAGGTCGACGATGGGCCCAACTGATTAAAGGTGCCGAACG |
| PamyB-R | | CAGTCTGGCCGTTTCCAAACATAAATGCCTTCTGTGGGGTTTATTGT |
| laeA-F | | ATGTTTGGAAACGGCCAGACTG |
| laeA-R | | TCAGTTCGCAGGTTTCCGTGCT |
| TagdA-F | | AGCACGGAAACCTGCGAACTGAAGGAAGCGTAACAGGATAGCCT |
| TagdA-R | | GGCGCGTTCTCGAGGAAGTTGCTCTTATTGCTTGTTTAGCTGTTAGGAC |
| pyrG-pMD19-R | | GGATCCTCTAGAGATGGGCCCCCCTTTTAGTCAATACCGTTACACAT |
| **Primers used for identification of TFs deleiton** | | |
| ΔprtT-ul-F* | GCACACTGGCACCACAAACGCT | |
| pyrG-inside-R | GATGATGTGTGCCCATTCGGAG | |
| pyrG-inside-F | CATCCGAACCCAACCATCTACG | |
| ΔprtT-dl-R** | CCTGATTCTTGCCGAGATGACG | |
| ΔamyR-ul-F | CAGGACAACTGGAGAAGCATCG | |
| ΔamyR-dl-R | TGTATGTCGGTGGGCTGTCTGA | |
| ΔcreA-ul-F | CCAACAATACGGGGCACGCACT | |
| ΔcreA-dl-R | CCAGCAAGTTGTTAGTCCAGGC | |
| ΔpacC-ul-F | TCGCCAACCTTTCCCATTCGTG | |
| ΔpacC-dl-R | AACCTCCCCTTTCCCATTGTCG | |
| ΔcpcA-ul-F | GTGACCTGCGAGTGACATTTCC | |
| ΔcpcA-dl-R | ATCCACCACTCTTTCGCTTCGG | |
| qGpdA-F | CTCTGCTCCTTCCGCTGAT | |
| qGpdA-R | ACCCTCAACGATGCCGAAC | |
| qCreA-F | ACATGAAGACGACGACGGTTATT | |
| qCreA-R | TGTGGTGGAGCGACAAATGA | |
| qPacC-F | GCCGCAAGAGCACAAACAA | |
| qPacC-R | GGCACCTCCACCGAACATC | |
| qAmyR-F | CTGCAGATGGAGTGGGCTAT | |
| qAmyR-R | TTGGTTGAGTCGGGTGAGG | |
| qPrtT-F | CTGCCAGACACTCCTTCAC | |
| qPrtT-R | TAAGCGGGTCCATAAGATTGA | |
| qCpcA-F | CTGGATACGCCTGGCTTCTT | |
| qCpcA-R | GAGTGCTTGGTGCCCTGACG | |
| **Primers used for functional identification of ATAC-seq instances.** | | |
| cpcA1-1F | TCGCCGGCTTTGCTGGCCCTATGACTCAttaattaatataaatactggcaagggatgcc | |
| cpcA1-2F | cggtattgactaaaagggTTGGGTCTGACAGCCAATCGCCGGCTTTGCTGGCCCT | |
| cpcA2-1F | TGCAAATTAGGGCAGCGCTAAACCCAAAttaattaatataaatactggcaagggatgcc | |
| cpcA2-2F | cggtattgactaaaagggATCTGACTCAGCAAATGCAAATTAGGGCAGCGCTAAAC | |
| M7A-1F | GAGTTGAATTTGCACCCGCTGACTCAGCttaattaatataaatactggcaagggatgcc | |
| M7A-2F | cggtattgactaaaagggTTCTCTTGTCCGCCTTTGAGTTGAATTTGCACCCGCTG | |
| M7B-1F | CATTTTTTCCTTCTTTTTTTACCCTCGCttaattaatataaatactggcaagggatgcc | |
| M7B-2F | ggtattgactaaaagggGGCTGACTCAGACGCGCGCATTTTTTCCTTCTTTTTTTACCC | |
| M5A-1F | AAAATAAGATCATTTTCGGCGGTCCCGCttaattaatataaatactggcaagggatgcc | |
| M5A-2F | cggtattgactaaaagggTGAATGCCTGAGGCGGAAAAAATAAGATCATTTTCGGCG | |
| M5B-1F | ATACAATTTTCCAAGCATTGCAAATGGCttaattaatataaatactggcaagggatgcc | |
| M5B-2F | cggtattgactaaaagggTCCTGCCTGAGGCATACAATTTTCCAAGCATTGCAAATG | |
| M5C-1F | GCAACAAAGAAGCCAGCTACACGCTGAttaattaatataaatactggcaagggatgcc | |
| M5C-2F | cggtattgactaaaagggGCTCTCCTTATGCCTGAGGCAACAAAGAAGCCAGCTACAC | |
| CCA4-1F | CCGACCAATCGACTGACCAGCTAGTAGttaattaatataaatactggcaagggatgcc | |
| CCA4-2F | cggtattgactaaaagggCATCCCTACCGACCAATCGACTGACCAG | |
| CCA5-1F | CCCGGCAATCCGACATCGGCCGGGGAGttaattaatataaatactggcaagggatgcc | |
| CCA5-2F | cggtattgactaaaagggGACCAATCAAATGCCCGGCAATCCGACATCGG | |
| CCAI-1F | gactaaaagggttaaTATGTTTCGAGCTCAGATTTAGT | |
| CCAI-2R | ttgccagtatttatattaattaaCTACAACAAACGACACGCCAGTC | |
| CREA1-1F | GGGGAAATTGCGATGAATCCACCAGCGCAAGCCGATAGCGGCGAAGAT | |
| CREA1-2F | cggtattgactaaaagggTGTCGGGCCGGGCCGGGGGAAATTGCGATGAATCCAC | |
| CREA2-1F | GATGCAATTTCTTTCAATTCCCGGGGATTCGCCGTCCAAGCCGATAGCGGCGAAGAT | |
| CREA2-2F | cggtattgactaaaagggCACTGGCCTGGGATGCAATTTCTTTCAATTCCCG | |
| CREA3-1F | GCTGGGGTTTCTCCGTGAAGGACTTGGGCTTGGAAGCCGATAGCGGCGAAGAT | |
| CREA3-2F | cggtattgactaaaagggCGTGATGTGGACGCTGGGGTTTCTCCGTGAAG | |
| CREA4-1F | GTGCCGACTTTCTCACGGTGTAAGCCGATAGCGGCGAAGAT | |
| CREA4-2F | cggtattgactaaaagggGGTAGCCTGGAGGTGCCGACTTTCTCACGGTG | |
| AREA1-1F | GGCGATAGTTATTTTATTTTTTGAGTCACTGGAACTGCCAAGCCGATAGCGGCGAAGAT | |
| AREA1-2F | cggtattgactaaaagggGGTCGGCGATAGTTATTTTATTTTTTGAG | |
| AREA2-1F | CATGCAAATCCGTAAATCCGATAATTTCATACCCAGCAAGCCGATAGCGGCGAAGAT | |
| AREA2-2F | cggtattgactaaaagggTCGTTGTGTAAAACATGCAAATCCGTAAATCCGAT | |
| AREA3-1F | GAGCCCGATAATCAATCTCCTTACGTTAAAAAGCCGATAGCGGCGAAGAT | |
| AREA3-2F | cggtattgactaaaagggGCCCTCCCCGAGCCCGATAATCAATCTCC | |
| prtT1-1F | TTTCCGTCGGCTCAGCGTTTTGTCCTCCttaattaatataaatactggcaagggatgcc | |
| prtT1-2F | cggtattgactaaaagggGGCGCCTTGAGACGCTTTCCGTCGGCTCAGCGTTTTG | |
| prtT2-1F | GATCCCGGCGATCGCCGTCGGACTCCGGttaattaatataaatactggcaagggatgcc | |
| prtT2-2F | aacggtattgactaaaagggCAACAACGCGATCCCGGCGATCGCCGTC | |
| prtT3-1F | CGGGATTCGAGTCTTTCTATAGAAGCAttaattaatataaatactggcaagggatgcc | |
| prtT3-2F | aacggtattgactaaaagggGAACCAGATACTCCGACGGGATTCGAGTCTTTCTATAG | |
| prtT4-1F | CCACTTTTTGCCCTCGCCCGACGGAGCTttaattaatataaatactggcaagggatgcc | |
| prtT4-2F | aacggtattgactaaaagggCTTGTTTACAATGAAGCCCACTTTTTGCCCTCGCCCG | |
| amyR1-1F | GATCCCCGATAATTGACCGAGATGGGACttaattaatataaatactggcaagggatgcc | |
| amyR1-2F | aacggtattgactaaaagggAAGCCGATAGCGGCGAAGATCCCCGATAATTGACCGAG | |
| amyR2-1F | CGGGAATTGAAAGAAATTGCATCCCAGttaattaatataaatactggcaagggatgcc | |
| amyR2-2F | aacggtattgactaaaagggGGACGGCGAATCCCCGGGAATTGAAAGAAATTGCATC | |
| amyR3-1F | GCGGAAATTTAAAGGGATTAATTTCCACttaattaatataaatactggcaagggatgcc | |
| amyR3-2F | aacggtattgactaaaagggAGAGTGACTAGGGGCGGAAATTTAAAGGGATTAATTTCC | |
| amyR4-1F | CTCAATTTCCCCGAGGATACGGACTATTttaattaatataaatactggcaagggatgcc | |
| amyR4-2F | aacggtattgactaaaagggTTCCTCCGCAATCCTCAATTTCCCCGAGGATACG | |
| bHLH1-1F | GTAATGGCGTCACGTGATGTAGGCttaattaatataaatactggcaagggatgcc | |
| bHLH1-2F | aacggtattgactaaaagggGCTATTTGTAATGGCGTCACGTGATGTAG | |
| bHLH2-1F | TCACGTGATTAACGACCTTGGAAGCGGGttaattaatataaatactggcaagggatgcc | |
| bHLH2-2F | aacggtattgactaaaagggCACTGGTTCAAAGTACATCACGTGATTAACGACCTTGG | |
| CCAAT1-1F | ATAAAATTTGGAGCCAATCATCCAGGGCttaattaatataaatactggcaagggatgcc | |
| CCAAT1-2F | cggtattgactaaaagggCAAAAGCTTACTTGCATAAAATTTGGAGCCAATCATCCAG | |
| CCAAT2-1F | AGTGAATTTGTGGCGGGCGGTGAAGCCCttaattaatataaatactggcaagggatgcc | |
| CCAAT2-2F | aacggtattgactaaaagggTCCAATCAACGACAGTGAATTTGTGGCGGGCGG | |
| CCAAT3-1F | CAAAATAATGAACCGTGATTGGGTGTTGttaattaatataaatactggcaagggatgcc | |
| CCAAT3-2F | acggtattgactaaaagggCCTTGGTGTAGCTTGCCCAAAATAATGAACCGTGATTGGG | |
| CORE-F | aacggtattgactaaaagggttaattaatataaatactggcaagggatgcc | |

* ul means the upstream localization of deletion TF.

** dl means the downstream localization of deletion TF
